# Recent Advances in Therapeutic Peptides: Innovations and Applications in Treating Infections and Diseases

**DOI:** 10.1021/acsomega.5c02077

**Published:** 2025-04-23

**Authors:** Deepshikha Sharma, Isha Dhiman, Swarnali Das, Deepak Kumar Das, Devlina Das Pramanik, Sandeep Kumar Dash, Arindam Pramanik

**Affiliations:** † Amity Institute of Biotechnology, Amity University, Noida, Uttar Pradesh 201301, India; ‡ Department of Physiology, University of Gour Banga, Malda, West Bengal 732103, India; § Department of Chemistry and Nanoscience, GLA University, Mathura, Uttar Pradesh 281406, India; ∥ School of Medicine, University of Leeds, Leeds LS97TF, United Kingdom

## Abstract

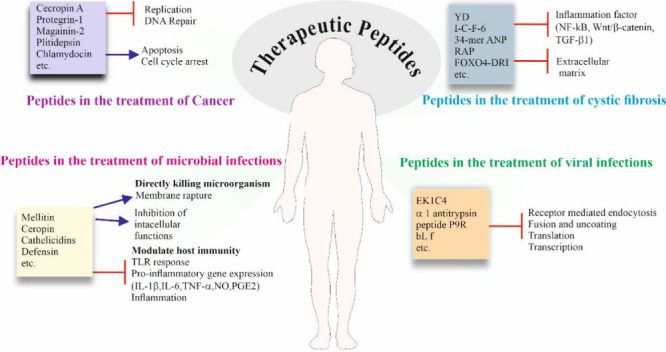

Peptides have become a powerful frontier in modern medicine, offering a promising therapeutic solution for various diseases and advancing rapidly in pharmaceutical development. These small amino acid chains, with their innovative design, have attracted significant attention due to their versatility and high receptor specificity, which minimizes off-target effects, along with enhanced therapeutic efficacy, biodegradability, low toxicity, and minimal immunogenicity. They are being explored for use in several clinical domains, like metabolic diseases, immunomodulation, and cancer. Furthermore, antimicrobial peptides (AMPs) have grown to be a promising strategy to combat the worldwide challenge of antibiotic resistance, demonstrating promising results against multidrug-resistant organisms. Both natural and engineered peptides have been discovered and investigated, whereas numerous others are progressing toward clinical trials in a number of therapeutic domains. Recent improvements with surface modification, such as peptide engineering, peptide cyclization, PEGylation, and the utilization of synthetic amino acids to enhance their pharmacokinetic profiles and overcome the inherent disadvantages of these peptides have made it possible for the area to continue to advance. Moreover, their therapeutic potential has been further enhanced by innovative delivery methods, such as self-assembling peptides, nanocarriers, and alternate routes of administration. This Review critically states the potential of peptides as versatile therapeutics along with their modifications and advancements to drive the significant progress to treat infections and chronic diseases, along with their potential benefits and challenges.

## 1 Introduction

Peptides, short sequences of amino acids, usually consist of 2–50 residues. The therapeutic application of bioactive peptides is anticipated to expand significantly in the near future. Current insights highlight their growing relevance in consumer skincare and cosmetic dermatology, particularly for their wrinkle-smoothing effects analogous to botulinum toxin and their association with collagen stimulation.^1,2^ It has been acknowledged that milk proteins are significant sources of peptides with biological activity. Following their release, these peptides have properties like antibacterial activity, antiviral activity, anticancer activity, therapeutic effects for cystic fibrosis and other interesting biological processes, such as opiate, antihypertensive, immunomodulatory, antithrombotic, or mineral-carrying properties.^3−5^ Insulin was the first peptide that was used therapeutically to treat type 1 diabetes in 1922.^6^ This event marked a significant advancement in the beginning of peptide-based therapies. These peptides represent a unique class of pharmaceuticals that fall between very small molecules and proteins. In the modern world, peptides have been used for several applications.

Regular and prolonged use of antibiotics has been associated with bacterial resistance to drugs and mutations, which has become a global health concern. However, peptides have been demonstrated as an alternative new generation of antibiotics. Peptides outperform conventional antibiotics in terms of effectiveness.^5,7,8^ Their broad-spectrum antibacterial, antifungal, and antiviral properties demonstrate their superiority over traditional antibiotics.^7,8^ Additionally, they are potent, having a low bactericidal concentration, rapid cell-killing effect, and effectiveness against a broad range of bacterial strains, including multidrug-resistant ones. Peptides are significantly superior to tiny chemical molecules in terms of potency, tolerability, specificity, and less frequent side effects (as amino acids are the end components of breakdown).^9^

Similarly, peptides also have potential anticancer activity. In cancer cells, they cause cell membrane rupture. A peptide’s potential to rupture membranes is dependent on several physicochemical characteristics, including the peptide molecules’ arrangement, net positive charge, structural conformations, and hydrophobicity in membranes; moreover, peptide concentrations, self-assembly, and membrane composition of cells are also important factors.^10^ Likewise, antiviral peptides (AVPs) have been identified to have therapeutic and preventative benefits against recognized coronaviruses. Potential AVP therapeutic targets can be found on the virus (such as E-protein and S-protein, which prevent viral binding) or on the host cell surface, such as angiotensin-converting enzyme 2 (ACE2) and transmembrane serine protease 2 (TMPRSS2).^11^ This Review summarizes a few recent studies on peptides for their diverse application in treating infections and diseases.

## 2 Anticancer Peptides

The preference of anticancer peptides (ACPs) is more for the cancer cells, which may be due to their structural and compositional variations of cell membranes over healthy cells. Having high concentrations of cholesterol in normal cells functions as a shield to control cell fluidity and prevent cationic ACPs from entering the cell or passing through. On the other hand, because their cell membranes contain less cholesterol than healthy ones, most malignant cells have more fluidity in their membranes, which could make them more vulnerable to ACPs. Additionally, cancer cells have an abundance of microvilli compared to healthy cells, the presence of which with uneven sizes and shapes affects cell adhesion and facilitates specific interactions among cancer cells and ACPs. Moreover it can also regulate the communication between cancer cells and their surroundings along with increased surface area for the binding and interactions with ACPs. Consequently, the enhanced selectivity and the ability of cell-killing of ACPs against the cancer cells may be caused by distinct cell membrane compositions, as well as the larger surface areas and greater fluidity of cancer cells over normal cells.

Alternatively, the mechanism of ACP activity has been explained by various mechanisms for the disruption of the membrane, such as the carpet model, the toroidal-pore wormhole model, and the barrel-stave model. The carpet model explains how negatively charged phospholipids of membranes associate with positively charged ACPs through electrostatic interactions in the outermost layer. As a result, ACPs align parallel with the cell membrane and envelop the cell like a carpet avoid to being entangled in lipid bilayers.^12^ However, peptides undergo molecular conformational changes upon reaching a threshold concentration. Their rotation, insertion into the membrane, and formation of micelle aggregates through hydrophobic interactions result in membrane fragmentation.

According to the barrel-stave model, the ACPs physically interact with hydrophilic segments on the cell membrane surface to attach themselves there first. The monomer peptide then experiences an alteration of structure and forms supramolecular self-assembly aggregate to form stave-like structures and transmembrane channels in the lipid bilayer.^13^ Peptide insertions provide a hydrophilic conduit through which the hydrophobic component of the bilayer is evacuated. More peptide molecules can enter and expand the size of the channel after it has developed. Furthermore, the integrity of the cell membrane is impaired as a result of the physical connection among ACPs and cancer cells. As of now, A lamethicin is currently the only ACP that kills cancer cells by using the barrel stave model.

The toroidal pore model describes the interactions of ACP along with cell membranes, which take place throughout two stages. First and foremost, at low concentrations, the peptide remains in its inactive form and aligns with the bilayer membrane. Subsequently, at certain concentrations, it changes into the active form and integrates into the membrane perpendicularly, which causes the bilayers to become irreversibly unstable by creating a pore structure that resembles a toroid.^14^ More ACPs may be able to enter the cell’s intracellular area through the produced toroidal pore. Numerous instances currently exist, and cecropin A, magainin-2, and protegrin-1 are examples of ACPs that use this method to destabilize cell membranes.

Many naturally occurring ACPs with anionic, cationic, and neutral characteristics have recently been identified in an array of organisms, like bacteria, fungi, yeast, plants, marine life, and bovine.^15^ Jang et al. studied bioactive peptides (DFHING, GLSDGEWQ, FHG, and GFHI) produced from bovine meat, which showed cytotoxicity in cancer cells.^3^ While the peptide of GLSDGEWQ greatly restricts the proliferation and expansion and of gastric adenocarcinoma (AGS cell line), GFHI shows the most toxic effect toward the human breast cancer cell line (MCF-7) and may also decrease the survival of the AGS cells in a a way that is dependent on concentration.

In the last few decades, the increased desire for naturally occurring peptides derived from dietary sources with anticancer effects has been noticed. For instance, the HVLSRAPR peptide isolated from *Spirulina platensis* hydrolysates showed cell-specific cytotoxicity in colorectal cancer cells (HT-29) but minimum inhibition of normal liver cell proliferation.^16^ Similar to this, a variety of peptide segments, including lunasin, GLTSK, LSGNK, GEGSGA, RKQLQGVN, MTEEY, and MPACGSS, are present in the hydrolysate of soybean protein and can have different antiproliferative effects on HT-29 cells.

New approaches to peptide stability and distribution create new opportunities for developing peptide therapies that can specifically target critical proteins involved in carcinogenesis. One such target is the proliferating cell nuclear antigen (PCNA). Cancer cells have high levels of PCNA expression, which is essential for cellular growth. It was discovered that R9-Peptide with 9 arginine residues (RRRRRRRRR) specifically inhibits the proliferation of cancer cells rather than nonmalignant or normal cells. It was discovered that PCNA’s interaction with its binding partners is disrupted by R9-Peptide. Furthermore, it was observed that the peptide interacted with the p66 subunit of DNA polymerase (POLD3).^17^ Studies using immunofluorescence microscopy revealed that R9-Peptide interfered with the connections between PCNA-FEN1 and PCNA-LIG1 during DNA replication. R9-Peptide therapy of cancer cells caused cell cycle arrest, DNA damage, and apoptosis as well as halted replication forks. R9-Peptide has shown therapeutic potential by preventing the formation of xenograft tumors derived from neuroblastoma cell lines and triple-negative breast cancer in mice.^18,19^

Enzyme-instructed self-assembly (EISA) peptides are short peptide sequences that undergo self-assembly into nanostructures when triggered by specific enzymes. The assemblies are the source of EISA’s cancer-inhibitory effect, and the peptides’ capacity to self-assemble regulates EISA’s anticancer action, as documented which looks at comparison in six different substrates of EISA. Results indicate that the anticancer effects of these precursors correlate with their capacity for self-assembly, independent of the regiochemistry and stereochemistry of their tetrapeptidic core.^20−23^ Moreover, the peptide assemblies that arise from EISA damaged plasma membranes and cytoskeletons, which resulted in cell death.

**Figure 1:**
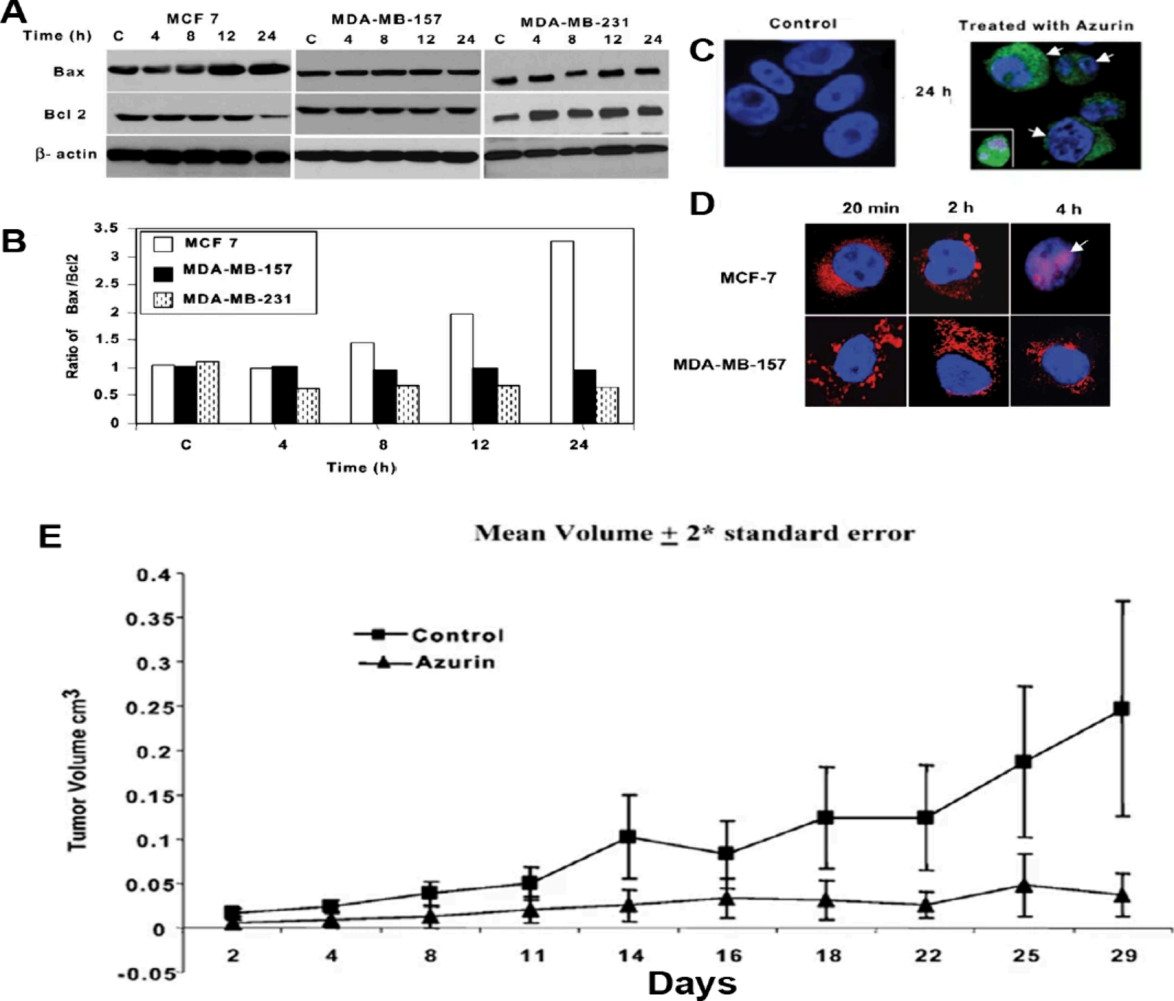
(A, B) Impact of the treatment with azurin on the expression of Bax and Bcl2 in MCF-7, MDA-MB-157, and MDA-MB-231 cells. During the treatment, the levels of Bax in MCF-7 cells improve, while those in Bcl2 decline. The levels of both proteins in MDA-MB-157 cells did not change significantly. Furthermore, Bax expression varies in MDA-MB-231 cells, decreasing at 8 h and then increasing at 12 and 24 h. In contrast, Bcl2 expression increases at 8 and 12 h but slightly decreases at 24 h. (C) Cleaved caspase-7 was detected by in situ immunodetection in MCF-7 cells after azurin treatment and was absent from cells treated with PBS for 24 h (control); however, a significant level of cleaved caspase-7 staining (fluorescing green) could be observed in cells treated with azurin after 12 and 24 h. Nuclei were stained blue with DAPI. Arrows mark the apoptotic cells. (D) Representative confocal microscopic images for subcellular localization of azurin after microinjection of Alexa Fluor 568-labeled azurin (fluorescing red) to MCF-7 cells and MDA-MB-157 cells. After 2 and 4 h of treatment, azurin shows nuclear localization in MCF-7 whereas in p53-negative MDA-MB-157 it shows cytoplasmic localization. Azurin shows cytotoxicity in MCF-7 but not significant cell death in p53-negative MDA-MB-157 and MDA-MB-231 cells. (E) Azurin shows significant tumor growth inhibition *in vivo* in MCF-7 xenografts. Adapted with permission from ref (25). Copyright 2025 Springer Nature.

*Pseudomonas aeruginosa* secretes a redox protein called azurin, which contains copper,^24^ Leu50-Asp77 makes up the p28 segment of the azurin protein. Azurin and p28 can penetrate cell membranes while also preventing growth or inducing apoptosis in several types of cancer cells. Azurin and p28 primarily use the p53 signaling pathway to inhibit cell division or cause apoptosis. It has been shown that proapoptotic genes such as Bax and Bcl-2 are expressed when P53 is stabilized by azurin/p28 and enters the nucleus.^25,26^ MCF-7 breast cancer cells treated with 53 μM azurin for 72 h showed 20% cell survival after treatment (Figure 1).^25^ Similarly, 44% cell death was observed in ZR-75-1 breast cancer cells after 72 h of treatment with 100 μM p28.^27^ Yamada et al. reported that azurin and p28 can effectively stop the formation of tumors in mouse models, such as Dalton’s lymphoma mice, 4T1 breast tumor mice, and MCF-7 human breast cancer xenograft on mice.^27−29^

Azurin and p28 can also act as cancer-targeted drug carriers because of their selectivity toward cancer cells. To increase their efficacy, several anticancer proteins and peptides were fused with azurin and p28. NRC peptide and apoptin exhibit enhanced anticancer activity against breast cancer cells upon fusion with p28.^30,31^ Shahbazi et al.^32^ demonstrated that when p28 and HPV16 E7 protein were fused, the resultant fusion protein activated the immune system and targeted cervical cancer cells effectively. Additionally, other cargos, including liposomes and nanoparticles, can be coupled to p28 to facilitate the release and distribution of drugs specifically targeted to cancer cells.^33,34^ Bernardes et al. observed that the coadministration of azurin increase the susceptibility of A549 lung cancer cells toward gefitinib and erlotinib.^35^ A similar outcome was observed in cases of cervical cancer (HeLa cells), breast cancer (MCF-7 cells), and colon cancer (HT-29 cells) to doxorubicin and paclitaxel^36^ when administrated with azurin. Yamada et al. discovered that using p28 in combination with antimitotic and DNA damage drugs increases their efficacy in various cancer cells.

**Figure 2:**
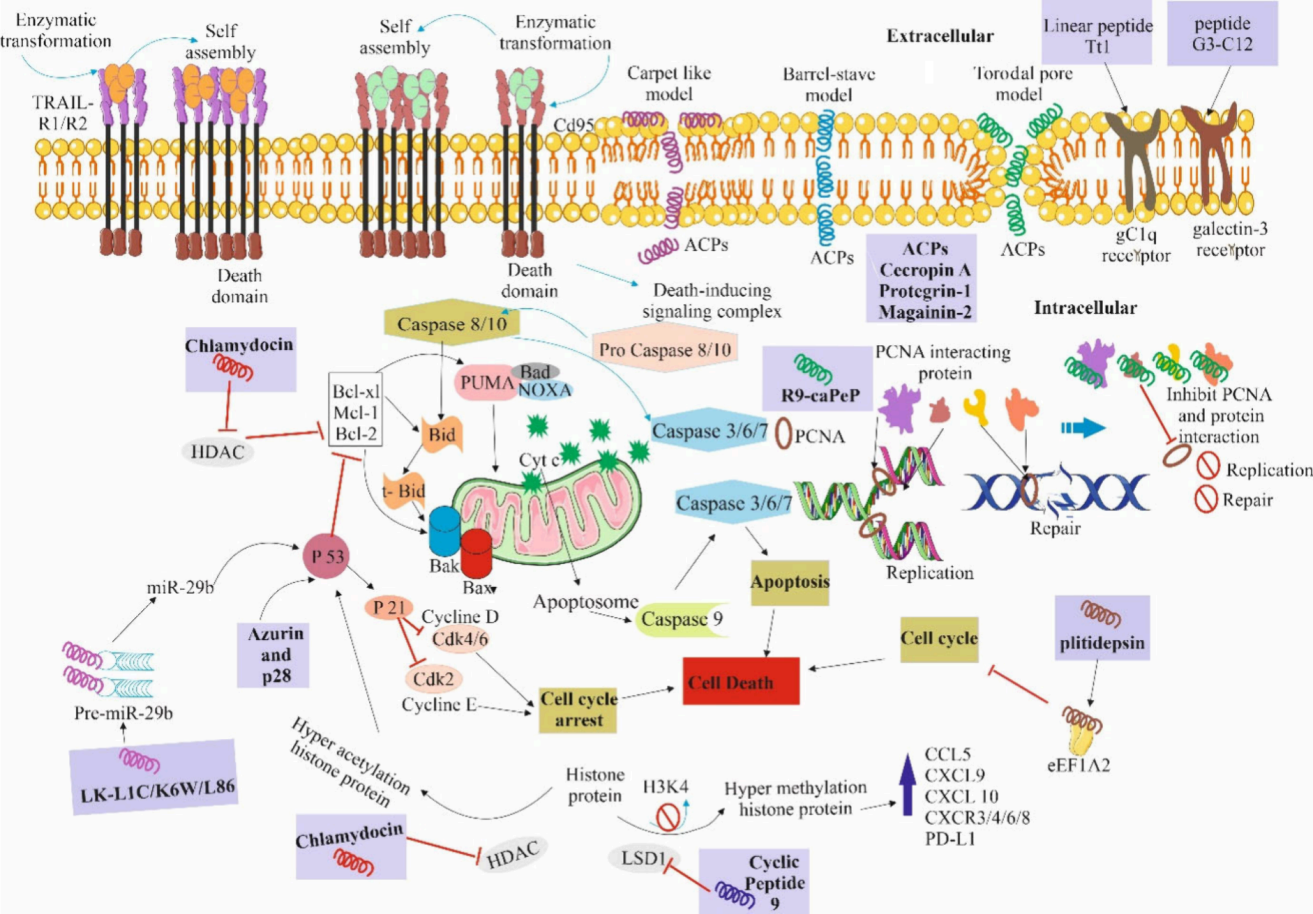
Schematic diagram of the anticancer mechanism of ACPs. ACPs target cancer cells through membrane disruption via the carpet-like model, barrel-stave model, and toroidal pore model. Additionally, peptides selectively interact with cancer cells and lead to cell death through cell cycle arrest, blocking DNA replication and DNA repair processes. Some peptides activate the P53 gene, which induces apoptosis by in extrinsic and intrinsic pathways, as well as activate p21, which is a cyclin-dependent kinase inhibitor that arrests the cell cycle. Peptides also can bind with PCNA and disrupt its function in DNA replication and repair, leading to cancer cell death. Some parts of the figure were drawn by using pictures from Servier Medical Art. Servier Medical Art by Servier is licensed under a Creative Commons Attribution 4.0 Unported License https://creativecommons.org/licenses/by/4.0/.

Food-derived peptides can regulate noncoding RNA transcription in addition to altering DNA methylation and histone changes. In human gastric cancer cells, a peptide hydrolysate extract or peptide combination, the source of which is the soft-shelled turtle, is also used in traditional Chinese medicine as a functional food and alters the expression of 101 different microRNAs (miRNAs). Since several of the elevated miRNAs target oncogenes and have tumor-suppressive properties, the peptide could potentially be applied as a therapeutic anticancer peptide^37^ (Figure 2).

### 2.1 Breast Cancer

The potential of peptide-based vectors to selectively bind to overexpressed cell receptors has recently drawn interest in the fight against breast cancer. In cancer treatment, heat shock protein (HSP) gp96, a molecular chaperone found in cancer cell membranes, is often targeted.^38,39^ The gp96 N-terminal helix–loop–helix region is specifically recognized by the peptide p37 (LNVSRETLQQHKLLKVIRKKLVRKTLDMIKKIADDKY), which can interfere with the intramolecular helix–helix interaction, limit conformational variations of gp96 and disrupting its chaperoning function.^39,40^

On the surface of cancer cells and the cells associated with cancer, including active angiogenic endothelial cells, cancer associated fibroblasts, and cancer-associated macrophages (TAMs), the protein p32 is frequently overexpressed.^41^ Linear peptide TT1(AKRGARSTA) has been utilized to target protein 32, also known as a transmembrane gC1q receptor.^42^ So, TT1 has been conjugated on the surface of liposomes. Consequently, M2 primary human macrophages absorbed half of the total liposome nanovesicles. It has also been observed that compared to the unmodified liposome, the one with the linear peptide TT1 modification exhibited superior interaction with the 3D breast cancer spheroids.^43^

In a study done by Feng et al., peptide CK3 (CLKADKAKC) showed a greater efficacy for binding to MDA-MB-231 breast tumor cells as compared to 4T-1, MCF-7, and MDA-MB-435 breast tumor cells.^43^ Similarly, specific binding of the peptide G3-C12 (ANTPCGPYTHDCPVKR) to the 30 kDa galectin-3 receptor is linked to the growth and metastases of cancer cells.^44^ In a work performed by Kumar et al., the peptide G3-C12 was joined to *N*,*N*′,*N*″*N*‴-1,4,7,10-tetra-azacyclododecane (DOTA) via a peptide GSG linker that was radiolabeled with 111In to target breast cancer.^45^ The outcomes demonstrated that the G3-C12 peptide could effectively bind to galectin-3 expressing human breast cancer MDA-MB-435 cells.

Zamani et al. developed a nanoliposomal vaccination by combining the long multiepitope peptide E75 AE36 (Ac-CGGGKIFGSLAFLAAAGVGSPYVSRLLGICL) with the peptide PADRE (AKFVAAWTLKAAA) and assessed the immunogenicity of vaccine against mouse breast cancer.^46^ The outcomes demonstrated that this nanoliposomal vaccine increased IFN-γ production along with the response of CD4+ and CD8+ T cells. In HER2-positive breast cancer, a nanoliposomal vaccine containing P5 peptide containing 21 amino acids (ELAAWCRWGFLLALLPPGIAG) was covalently associated with the surface of the liposome.

In mice having a HER2-positive, tumor rat HER2/neu protein derivative P5, which is a CTL-specific peptide, can efficiently stimulate CTL responses,^47−50^ Monophosphoryl lipid A (MPL), an agonist of toll-like receptor 4 (TLR4), is one of the hydrophobic immune-stimulating compounds.^51,52^ Pan HLA-DR epitope peptide (PADRE) is one of the most effective compounds to trigger CD4+ responses in both humans and mice. According to recent research, when taken in combination with other vaccinations, PADRE can boost the vaccine potency and function as a strong immunogen.

P5, MPL, and peptide PADRE adjuvant demonstrated strong anticancer efficacy and successfully stimulated the CD8+ T cell immunological response.^53^ Clinical trials have been conducted on major peptide-based vaccines such as E75, GP2, AE37, P3, P4, P5, P7, P13, P14, and P15, as well as MUC1-KLH conjugate plus QS-21, MFP, and L-BLP25. While peptide-based vaccines in combination with anticancer medications have been used in late-stage cancers, peptide-based vaccine monotherapy has mostly targeted premalignant cancer.

### 2.2 Glioblastoma

Glioblastoma (GBM) is the tumor of a brain specified by the existence of distinct anaplastic cells encompassed by necrotic areas of the brain tissue. The tumor develops in astrocytes (star-shaped brain cells) that support the neurological system of the brain. There have been several therapeutic studies performed in GBM using peptide molecules. In this regard, three different categories of peptides that have been utilized for the specific administration of therapeutic drugs are cell-penetrating peptides (CPPs), peptides that target aberrant cell signaling pathways, and tumor-homing peptides,

#### 2.2.1 Tumour Homing Peptides

These peptides stick themselves specifically to receptors or proteins that are either overproduced or specifically exhibited on the cancer cell surface. Several peptides can also bind to cancer cells or tumor tissues, enhancing or opposing signal transduction pathways. Some examples of tumor homing peptides in the context of GBM are anigiopep-2 (ANG), chlorotoxin, and interleukin-13 receptor a2 (IL-13Ra2) targeting peptides.

Anigiopep-2 (ANG) is a synthetic peptide with 19 amino acids. One of its unique advantages is its ability to pass the blood–brain barrier (BBB). The low-density lipoprotein receptor-related protein (LRP-1) acts as a channel for its passage through the BBB. It has been utilized to deliver drug molecules to brain tumors. In a work done by Xin et al., polyethylene glycol-based nanoparticles (PEG-NP) packed with paclitaxel (ANG-PEG-NP-PTX) were modified using Angiopep-2.^54^*In vivo* model studies with ANG-PEG-NP-PTX revealed that the average survival period for these animals increased to 37 days compared to the other groups of mice given saline treatment (22 days), PEG-NP-PTX (30 days), or Paclitaxel (25 days), indicating that ANG-PEG-NP-PTX has a potential for therapeutic application of GBM.^55^

Chlorotoxin, a 36-amino acid peptide, is present in the venom of the deathstalker scorpion (*Leiurus quinquestriatus*)^56^ that can inhibit small-conductance chloride channels. Soroceanu et al. identified that surface-bound matrix metalloproteinase-2 (MMP-2) is typically not produced in brain tissue but is abundantly expressed in GBM. Chlorotoxin exhibits a high preference for surface-bound MMP-2.

A plasma membrane receptor called interleukin-13 receptor a2 (IL-13Ra2) is found in 75% of GBM but not in healthy brain tissue. Peptide-1 linear (Pep-1L), an IL-13Ra2-targeting peptide, was created as a molecular scaffold by Sattiraju et al. The team developed Pep-1L-conjugated paclitaxel particles and Pep-1L linked to cytotoxic α-particle-emitting radioisotopes to study its efficacy against GBM. It was observed from this study that these Pep-1L conjugated molecules had significant antiglioblastoma activity in *in vitro* models. From an *in vivo* mouse model study, these IL-13Ra2-targeted peptides tagged with a therapeutic increased the survivability of mice^57,58^ in addition to reducing tumor growth.

#### 2.2.2 Targeting Abnormal Cellular Signaling Pathways

These peptides can control oncogenic signaling pathways, which control the growth, evasion of apoptosis, and function of cancer cells. Consequently, with enhanced selectivity, precisely constructed peptides and their derivatives are able to be included in drug delivery systems to target many biochemical pathways within tumor microenvironments and enhance tumor therapy. Glioblastoma cells overexpress the voltage-dependent anion channel 1 (VDAC1), which is crucial for energy metabolism, controls mitochondria-mediated apoptosis through engaging with antiapoptotic proteins, and inhibits glioblastoma cells death.^59^ Shteinfer-Kuzmine et al. developed VDAC1-targeting peptides that inhibited the anti-apoptotic activities of these proteins, activated mitochondria-mediated pathways, and initiated apoptosis. In the orthotopic glioblastoma mouse model, it was observed that peptides based on VDAC-1 coupled to either CPP or the transferrin receptor significantly restricted tumor growth.^60^

Friedmann-Morvinski et al. created a peptide known as the NEMO-binding domain (NBD) to target NEMO. This peptide suppresses NF-kB activity and prevents NEMO from interacting with the IKK (IkB)–kinase complex. NBD treatment confirmed that one intriguing target for the therapy of glioblastoma is the NF-kB pathway, as it inhibited tumor growth in both mouse and human glioblastoma models *in vitro* and increased the survivability of mice from 30 days in the control group to more than 50 days.^61^

#### 2.2.3 Cell-Penetrating Peptides

Cell-penetrating peptides (CPP), short peptides with the capacity to cross cell membranes, are produced from insects, viruses, or mammals. Targeting drugs inside GBM is possible with the covalent coupling of these CPP peptides to different drug carriers, such as nanoparticles and liposomes. CPPs emerged from the transcriptional activator TAT protein of the human immunodeficiency virus type 1 (HIV-1), which was paired with polyamidoamine (PAMAM) dendrimers loaded with siRNA.^62^ This indicated an increased therapeutic impact of siRNA *in vivo*. Studies by Gupta et al.^63^ further supported the concept that TAT can deliver genetic material. Intracranial human brain tumor xenografts in nude mice could effectively transfer a plasmid encoding a green fluorescent protein (pEGFP-N1) via TAT-decorated liposomes. Further *in vivo* research showed *Antennapedia homeodomain*-derived penetrating peptide (Antp) and SynB1 CPPs, produced from natural peptides termed protegrins, have the therapeutic potential to treat glioblastoma by improving the drug’s transport across the blood–brain barrier.^64,65^ Although CPPs can deliver a wide variety of cargo into cells, their lack of specificity continues to raise concerns. Combining CPP-derived therapies with other targeted delivery methods, such as tumor-homing ligands, can increase their specificity and ensure effective and focused drug administration.

#### 2.2.4 Peptides as Epigenetic Regulators for Controlling Cancer

Epigenetic modifications are shifts in the phenotype and gene expression that do not affect the DNA sequence. Epigenetic regulation may become nonfunctional during the embryonic phase, potentially due to sperm cells experiencing increased oxidative stress, leading to congenital disorders including Hirschsprung disease and fragile X syndrome.^66−69^ Peptides can impact several aspects of epigenetic regulation. Despite this, epigenetic mechanisms also control the expression of endogenous peptides. Histone alterations, noncoding RNAs, and DNA methylation are prominent instances of epigenetic modifications. For instance, altering the patterns of DNA methylation in the promotor regions of peptide regulates the expression of vasopressin and natriuretic peptides upon the cessation of alcohol and tobacco use.^70−73^ Additionally, in cancerous tissue, peptides specific to cancer, like members of the trefoil factor family that play critical roles in oncogenic transformation, are expressed due to epigenetic mechanisms.^74,75^ Following epigenetic therapy with DNA methyltransferase (DNMT) and histone deacetylase (HDAC) inhibitor,^76,77^ cryptic long terminal repeat (LTR) transcripts of endogenous retroviruses (ERV) has been reported as a new class of treatment-induced nonannotated out-of-frame dsRNA transcripts (TINATs).^78^

Cryptic peptides can be synthesized or produced endogenously by cleaving nuclear proteins with protease enzymes, which allows the peptides to penetrate through both the nuclear and cytoplasmic membranes.^79,80^ These peptides play a role in the epigenetic control of aging and can improve health by inhibiting age-related increases in matrix metalloprotease production and caspase-dependent apoptosis.^81,82^

Lunasin is a 43-amino acid polypeptide produced from soybeans that has demonstrated strong anticancer properties and the ability to suppress core histone acetylation of H3 and H4.^82,83^ The peptide has eight negatively charged ‘Asp residues’ at its carboxyl-terminal end, which function as suppressors of the positively charged H3 and H4 acetylations. A 9-amino acid α-helical structure that directs and binds lunasin to the core histone proteins comes right before this sequence, as does the Arg-Gly-Asp (RGD) motif, which binds to the extracellular matrix and helps the peptide penetrate cells.^84^ This peptide has also been found to have beneficial effects on neurodegenerative illnesses such as amyotrophic lateral sclerosis (ALS) and Alzheimer’s disease (AD).^85^

The marine tunica *Aplidium albicans* is the source of a cyclic depsipeptide named plitidepsin (aplidin) . It binds to the eEF1A2 protein, through a variety of ways, and induces cell-cycle arrest, growth inhibition, and apoptosis. It also has pleiotropic efficacy on cancer cells and is thought to be a histone deacetylase (HDAC) inhibitor.^76,77^ The cyclic tetrapeptides represent a second class of peptide HDAC inhibitors. This category includes the following: FR235222, apicidin, chlamydocin, microsporins (A and B), azumamides (A–E), and trapoxin.^76,77,86^

Fungal metabolite chlamydocin is a cyclic tetrapeptide that has a potent HDAC inhibitory effect and causes hyperacetylation of histones H3 and H4, which halts the cell cycle in the G2/M phase and triggers caspase-3 to trigger apoptosis. Furthermore, it suppresses survivin, an apoptosis inhibitor that is only expressed in tumors.^87^

The chromatin-remodeling enzyme lysine-specific demethylase 1 (LSD1) transfers methyl groups from lysine at histone H3 position 4. Due to its significant role in cancer and its ability to silence tumor-suppressing genes when overexpressed, this enzyme is a desirable target for treatments.^88^ Forneris et al. generated a peptide with methionine in place of lysine at position 4; as a result, the value of *k*_i_ decreased from 1.8 to 0.04 μM.^89^

An amphiphilic peptide called LK-L1C/K6W/L8C binds to the pre-miR29b terminal loop area, maturing into miR29b, which then causes cancer cells to undergo apoptosis by stabilizing p53. When this peptide binds to pre-miR29b, it promotes complexation with the miRNA maturation enzyme Dicer and increases the level of production of miR29b. As a result, this peptide can increase the development of apoptosis within cancer cells by upregulating p53 and miR29b.^90^ One of the most effective miRNAs, miR-155 is overexpressed in many cancer types and inhibits apoptosis in human cancer cells. Pai et al. used peptide microarrays to identify two peptides that inhibit the maturation of Dicer-mediated miRNA-155 through upregulating miRNA-155 target genes and triggering caspase-dependent pathways, which promote the cell death. These peptide inhibitors attach to the apical stem-loop domain of the pre-miRNA, disrupting Dicer’s interaction site and decreasing Dicer-mediated processing. As a result, they may be used as novel treatments to treat a variety of cancer forms.^91^

### 2.3 Clinical Trials of Anticancer Peptides

A multitude of synthetic peptides and vaccines are presently undergoing clinical testing. When CIGB-300, a peptide-based inhibitor of casein kinase 2, combines with the cell-penetrating peptide trans-acting activator of transcription (TAT), it can stop casein kinase 2-mediated phosphorylation, which results in the death of cervical and nonsmall cell lung cancer cells.^92−94^ A GV1001 peptide vaccine was created based on hTERT (EARPALLTSRLRFIPK) and assessed in phase I/II clinical studies on patients with incurable pancreatic cancer. Research indicates that it possesses the ability to stimulate CD4+ and CD8+ T-cells, interface with specialized antigen-presenting cells, then destroy cancer cells or tissue.^95^ A personalized peptide vaccination was developed recently to enhance immune response by using unique peptides for every patient, serving as a revolutionary approach to cancer treatment. For instance, a phase II clinical trial evaluated 19 peptide variants chosen among 31 customized peptide vaccines in individuals with malignant breast cancer. Other peptides of gp100:209-217 (210M)/montanide ISA-51/imiquimod and E39 peptide/granulocyte macrophage colony stimulating factor vaccination plus E39 booster have been approved by the U.S. Food and Drug Administration to treat high-risk ovarian cancer and melanoma, respectively.^96^ (Table 1).

**Table 1:** List of Therapeutic Peptides with Anticancer Activity with Their Clinical Trail Stage^a^

phase	biological peptides	cancer types	outcome
early phase I	MUC-1 peptide vaccine	breast cancer	positive anti-MUC1 antibody responses
	HER-2/neu peptide vaccine	breast cancer	specific interferon-γ and IL-5 producing T-cell responses
	GAA/TT-peptide vaccine and poly-ICLC	astrocytoma, oligoastrocytoma, and glioma melanoma	GAA-specific T-cell responses
phase I	Gag:267-274 peptide vaccine	melanoma	Cytotoxic T-cell lymphocyte responses
	HPV16 E7 peptide-pulsed autologous DCs	cervical cancer	pulsed autologous DC immunotherapy
	LY6K, VEGFR1, VEGFR2	esophageal cancer	immune responses including LY6K, VEGFR1 and VEGFR2 specific T-cells
	antiangiogenic peptide vaccine	hepatocellular carcinoma	cytotoxic T-cell lymphocyte responses
	HLA-A*0201 or HLA-A*0206-restricted URLC10 peptides	nonsmall cell lung cancer	cytotoxic T-cell lymphocyte responses, antigen cascade, regulatory T-cells, cancer antigens and human leukocyte antigen levels
phase I/II	MAGE-3.A1 peptide and CpG 7909	malignant melanoma	cytotoxic T-cell lymphocyte responses
	VEGFR1–1084, VEGFR2–169	pancreatic cancer	cytotoxic T-cell lymphocyte responses
	HER-2/neu peptide vaccine	breast cancer	human epidermal growth factor receptor 2-specific T-cell response
phase II	gp100:209–217(210M), HPV 16 E7:12–20	melanoma	T-cell immunity
	WT1 126–134 peptide	acute myeloid leukemia	T-cell response
	G250 peptide	metastatic renal cells carcinoma	cytotoxic T-cell lymphocyte responses
phase III	PR1 leukemia peptide	leukaemia	immune response
phase IV	degarelix	prostatic neoplasms	binds to GnRH receptors

a Reprinted from ref (96). Copyright Chiangjong et al., which is under open access licence CC BY-NC-ND 4.0.

## 3 Fibrosis

Fibrosis is the abnormal and excessive accumulation of fibrous connective tissue in an organ or tissue, often as a result of chronic inflammation or injury. This process can disrupt normal tissue architecture and impair organ function, commonly seen under conditions such as liver cirrhosis, pulmonary fibrosis, and cardiac fibrosis. Alteration of sweat, digestive fluids, and mucus production are all associated with cystic fibrosis (CF). The cystic fibrosis transmembrane conductance regulator (CFTR) gene is altered in the individuals who suffer from cystic fibrosis, accounting for approximately over 30 000 individuals in the United States and around 89 000 individuals worldwide. The reduced function of the CFTR protein is associated with decreased life expectancy and multiorgan dysfunction. The majority of mucus in healthy individuals is made up of glycoproteins, which serve as a physiological barrier to protect the body from toxins and infections. However, in CF lungs, the damaged ciliated epithelium does not remove the mucins and become excessively produced and oversecreted in response to the inflammation and infections of respiratory tract. Additionally, the CF mucus has a higher concentration of actin released from necrotic cells and DNA produced by pathogens and necrotic neutrophils, which increases the mucus’s viscosity and adhesiveness and reduces mucociliary clearance.^97−99^ Mucus plaques become thicker and more viscous, which depletes them of oxygen. This creates an environment favorable for bacterial infections and the subsequent development of a biofilm.^100^ Resistance to antibiotics and the subsequent persistence of infections are caused by bacteria that evade defense mechanisms and have a competitive advantage when growing in biofilms. This is a significant difficulty in the treatment of cystic fibrosis (CF).

### 3.1 Peptides as Therapeutics for Fibrosis

Employing carriers such as nanoparticles (NPs) is one way to increase penetration and decrease retention in the mucus.^101^ Peptides are among the drugs that can be bonded to the surface of NPs or encapsulated within them. Applying muco-inert and electrostatically neutral polymers to the surface of NPs can potentially decrease both hydrophobic and electrostatic interactions.^101,102^ Leal et al. used a model of CF mucus to find novel mucus-penetrating peptides for diffusive transport by using phage libraries.^4^ 2.0 × 10^10^ randomly distributed heptagonal polypeptides with a flexible linker (GGGS) were genetically injected into the phages’ genomes to be expressed on phage surface proteins. This selection produced 30 phages, which were then separated, and their matching peptides were sequenced. Interestingly, these peptides were more abundant in Pro, Ser, and Thr amino acids than in the original library. These amino acids are the structural unit of mucin proteins, and their enrichment in them suggests that these peptides may disperse in mucus as a result of weak intermolecular interactions. Additionally, a significant portion of the detected sequences exhibited neutral charges and were hydrophilic, which further elucidated their enhanced penetration in CF mucus.^4^ Overall, effective inhaled administration of drugs, including peptides and proteins, that would not otherwise be appropriate to treat CF is made possible by altering the physical and chemical characteristics and the application of NPs to increase mucus penetration.

**Figure 3:**
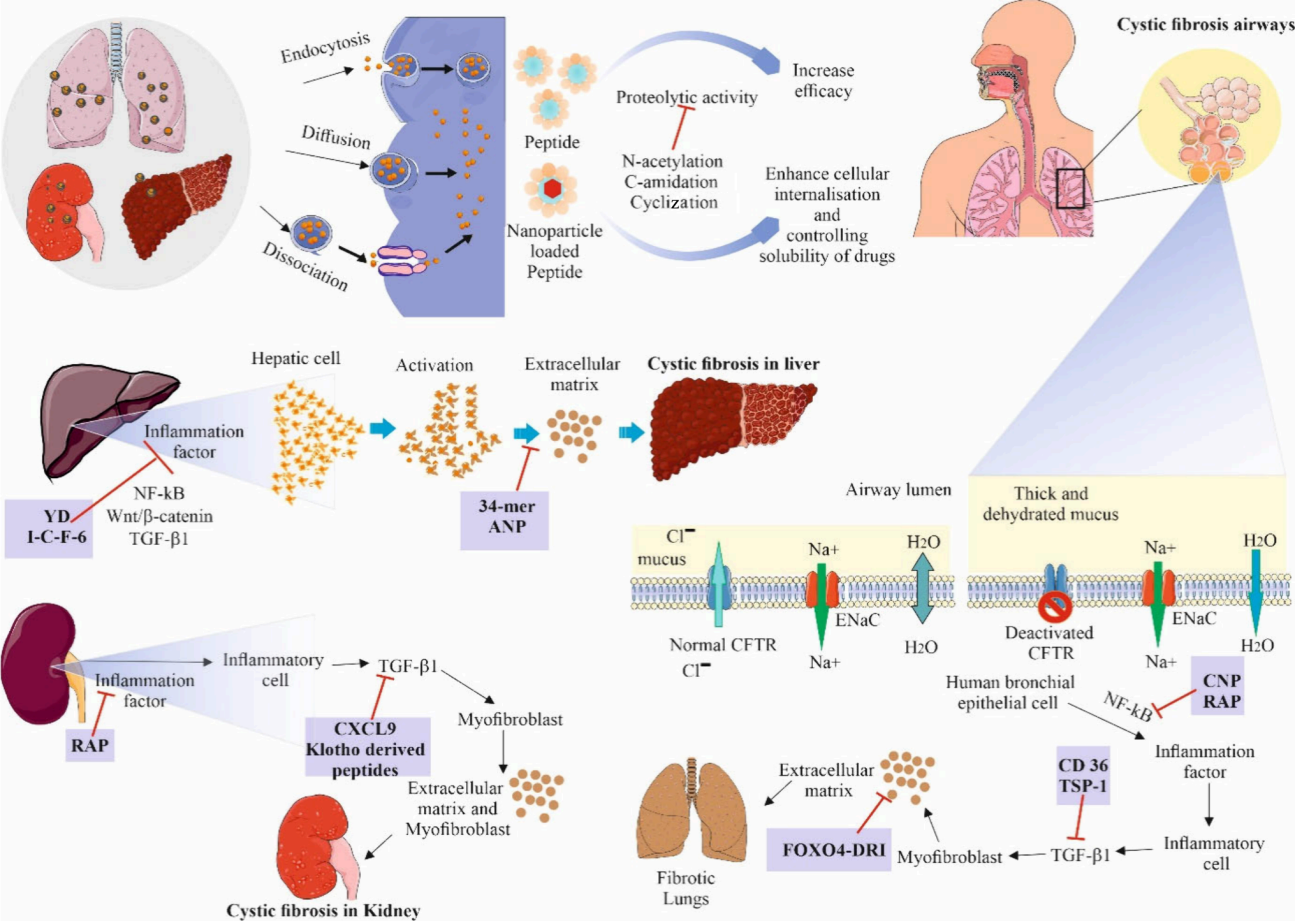
A representation of the role of peptides in treating fibrosis in airways, kidney, and liver. All the fibrosis types are controlled by peptides through different mechanisms. In case of all fibrosis types, peptides help to inhibit the inflammation factor NF-κB, which activates TGF-β1, and some peptide directly inhibit TGF-β1, which plays a crucial role in the development of fibrosis via activating myofibroblasts. Some peptides were also found to inhibit the extra cellular matrix, which leads to tissue scarring and impaired function in case of lung and liver fibrosis. Some parts of the figure were drawn by using pictures from Servier Medical Art. Servier Medical Art by Servier is licensed under a Creative Commons Attribution 4.0 Unported License https://creativecommons.org/licenses/by/4.0/.

Moreover, several studies have identified the development of peptide pharmaceutical antifibrosis therapies to serve as a scientific reference. During hepatic fibrosis, the efficacy to target miR-155 with CASP12 and lower inflammation was discovered through an isolated antimicrobial peptide YD from *Bacillus amyloliquefaciens* CBSYD1.^103^ Furthermore, another study identified that the herb *Carapax trionycis* contains several peptides having strong hepatoprotective effects and oligo-peptide I-C-F-6, which inhibits NF-κB and Wnt/β-catenin signaling to prevent HSC activation and reduce CCl_4_-induced liver fibrosis.^104^ Another study reported that the pigment epithelium-derived factor 34-mer peptide, an inherent antifibrotic factor, inhibits the platelet-derived growth factor receptor, thereby preventing hepatic stellate cell activation and liver fibrosis.^105^ Continuous intravenous atrial natriuretic peptide (ANP) injections prevented liver fibrosis by protecting hepatocytes and inhibiting stellate cell activation in DMD-induced liver fibrosis.^105^ Similarly, a peptide produced by CD36 inhibits the lung fibrosis that is induced by silica via reducing too much TGF-β1 activity.^106^ According to a report of another study, TSP-1 synthetic peptide has been shown to bind with CD36, preventing the TSP-1/L-TGF-1/CD36 complex from shielding mice from bleomycin-induced lung fibrosis.^107^ Another previous study reported that FOXO4D-Retro-Inverso peptide preferentially induces senescent cell apoptosis and inhibits the connection between FOXO4 and p53, specifically targeting myofibroblasts. It also reduces bleomycin-induced pulmonary fibrosis in mice.^107^ Murakami et al. also claim on their study that the CNP inhibits pulmonary inflammation along with the proliferation of cells, hence reducing bleomycin-induced lung fibrosis.^108^ Beside that renal interstitial fibrosis or kidney disease or inflammation was accompanied by an increase in renal NF-κB activation. According to a prior study, kidney fibrosis is reduced by the antioxidant peptide RAP, which is produced from rapeseed protein through the inhibition of MAPK/NF-κB signaling pathways.^109^ The study form Yuan et al. also suggested that a peptide derived from klotho reduces kidney fibrosis through concentrating on TGF-β signaling.^110^ Another study identified that glycosaminoglycan-binding peptide produced from human CXCL9 prevents renal fibrosis via targeting the GAG–protein interactions in renal fibrotic illness and allowed CXCL9 to have antifibrotic and anti-inflammatory effects (Figure 3).

#### 3.1.1 Enhancing Intracellular Absorption

The mucus that is thick and sticky in the airways of patients with CF is an obstacle for drugs to pass and perform their therapeutic function. The use of cell-penetrating peptides (CPPs), which enable the intracellular internalization of a variety of drugs, including biologicals, is one potential method to address the challenge. For example, penetratin is a protein found in *Drosophila melanogaster* that originates from the homeodomain of *Antennapedia*,^111,112^ 16 amino acids (RQIKIWFQNRRMKWKK) that correspond to the third helix of the *Antennapedia* homeodomain, were found to control intracellular absorption through structure–function studies. Several additional CPPs have been discovered in addition to TAT and penetratin. These are generally characterized by sequences made up of 5–30 amino acids that can penetrate biological membranes via either energy-dependent or energy-independent methods. These peptide sequences are now being used to deliver proteins, DNAs, siRNAs, peptides, and small drugs. It is possible to covalently conjugate these compounds to CPPs through chemical linkages, such as thioester or disulfide bonds, or by cloning and expressing fusion proteins later on. Nevertheless, these techniques have the potential to limit the biological activity of conjugated pharmaceuticals.. Another approach is to bind the drugs to the CPPs via hydrophobic or noncovalent electrostatic interactions, which will be valuable in preventing the breakdown of drugs by nucleases or proteases.^113,114^

#### 3.1.2 Restricting Proteolytic Cleavage

One of the main challenges in using peptides as therapeutics for CF is proteolysis. Using N-acetylation or C-amidation to stabilize a extremities of peptides is a possible way of preventing proteolytic degradation, in addition to shielding particular cleavage motifs throughout the entire peptide sequence.^115^ Another effective strategy for reducing peptide proteolysis is cyclization, which results in conformational restrictions that make it challenging for proteases to access and recognize cutting sites, since the binding of N and C termini fixes the mobile ends. Cyclization locks peptides in an active conformation, which increases their activity, because of these constraints. Colistin, a cationic polypeptide antibiotic, is the most renowned cyclic peptide that is used in CF therapy. However, early reports of significant toxicity led to its removal from treatment in the early 1970s.

#### 3.1.3 Controlling Aggregation and Solubility

Poor solubility causes proteins and peptides to aggregate, and these two processes are similar in their molecular mechanism when polypeptide chains break into unstructured globules.^116^ According to Guan et al., certain synthetic peptides can self-assemble to poloxamines to create compacted nanoparticles that are safe for administration via the lungs, as shown in CF model of mice.^117^ The results presented underline the advantages of peptide-loaded NPs for inhaled formulations and imply that the use of poly(*p*-phenylene ether) (PPE)/poly(*p*-phenylene oxide) (PPO) polymers could be a useful method to increase the solubility and delivery of peptides based drugs.

## 4 Antimicrobial Peptides

Long-term usage and misuse of standard antibiotics result in bacterial drug resistance, which poses an alarming threat to global health. Due to this reason, common antibiotics are slowly becoming ineffective. Among the alternatives, antimicrobial peptides (AMPs) could be potential candidates as next generation antibiotics for tackling drug-resistant microbes. The host defense peptides known as AMPs are mostly α-helical peptide molecules that are cationic (positively charged) and amphiphilic, *i.e.,* hydrophilic or hydrophobic in nature. The negatively charged bacterial cell membranes are susceptible to binding and interaction from these cationic AMPs, which can alter the electrochemical potential of the membranes, cause damage to the membranes, allow larger molecules like proteins to pass through, destroy the membranes and morphology of the cells, and ultimately cause the cells to die.^118,119^

**Figure 4:**
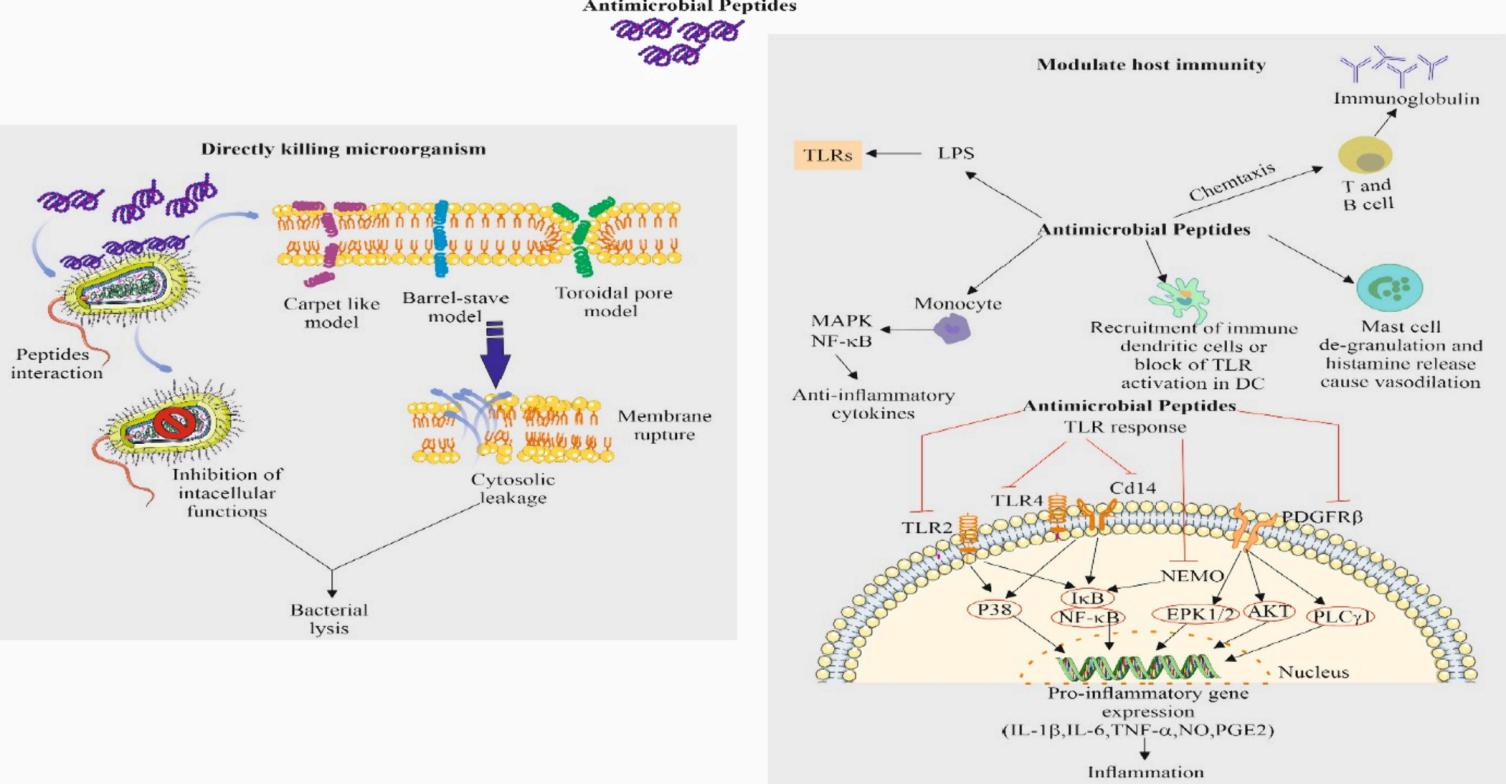
Mechanism of action of antimicrobial peptides to combat microbial infections. These antimicrobial peptides show a membrane-targeting mechanism through different modes of action, include carpet-like, barrel-stave and toroidal-pore models, leading to membrane rupture and cytosolic leakage. Besides that, some peptides alter the intracellular mechanism. Both pathways of these peptides lead to bacterial cell lysis. Additionally, some antimicrobial peptides also can modulate the immunity of host by mainly altering the inflammatory reaction caused by LPS. These peptides also can bind with TLRs and CD14 to inhibit the release of immune factors, which regulate the immune function of immune cells. These peptides also can work on different sites like monocytes, dendritic cells, mast cells, and T and B cells. Some parts of the figure were drawn by using pictures from Servier Medical Art. Servier Medical Art by Servier is licensed under a Creative Commons Attribution 4.0 Unported License https://creativecommons.org/licenses/by/4.0/.

Due to several advantages, natural AMPs, have attracted a lot of interest as antimicrobial medications in recent years. They are characterized by a broad variety of activities, a quick mode of action, relative selectivity toward their targets (microbial membranes), and, most importantly, a low frequency of selection of resistant strains.^120^ Here, some of the natural AMP such as mellitin, cecropin, cathelicidins, defensin, magainin, dermaseptins, eumenitin, and HistaiN are discussed for their recent antimicrobial studies (Figure 4).

### 4.1 Mellitin

Melittin is a strongly cationic peptide, originally isolated from the venom of the European honey bee *Apis mellifera*, and is reported to possess anti-inflammatory activity.^121,122^ It is made up of 26 amino acid residues (GIGAVLKVLTGLPALIWIKRKQQ). The melittin peptide is linear and amphipathic, which improves its capacity to cause bacterial and eukaryotic cell membranes to permeabilize.^123^

#### 4.1.1 Mellitin against *Leishmania*

Numerous studies have reported a beneficial effect of melittin against the disease leishmaniasis. One recent study demonstrated the activity of melittin against *L. donovani* promastigotes with an IC_50_ less than 1.5 μM. Recently, another study reported that melittin showed good activity on *L. major* and *L. panamensis* promastigotes, with IC_50_ values of 100 μg/mL and 74.01 μg/mL respectively.^124^ Pereira et al. showed the effectiveness of melittin against *L. infantum* in both stages, *i.e.*, promastigotes and amastigotes.^121^

#### 4.1.2 Mellitin against *Trypanosoma*

**Figure 5:**
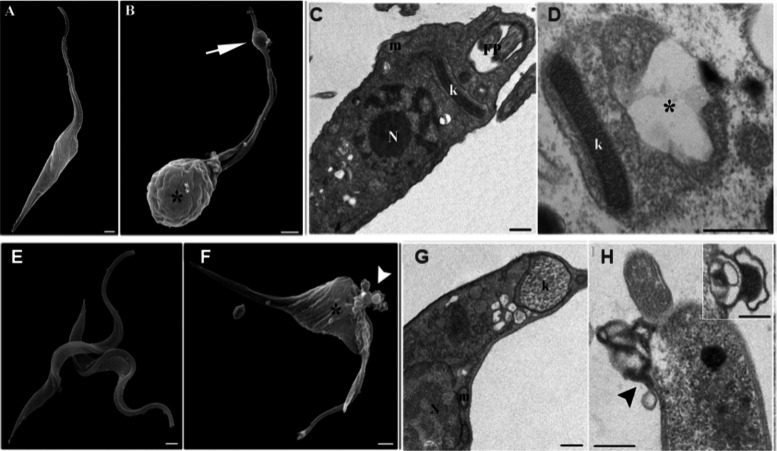
Ultrastructural evaluation of epimastigotes and trypomastigotes treated with melittin. (A, D) The usual elongated cell body and normal nuclear morphology (N), kinetoplast (k), mitochondria (m), and flagellar pocket (FP) were all present in the untreated epimastigote parasites. (B, C) Altered flagellar morphologies with cracks, swelling structures, and a broken appear (arrows), as well as enlarged and aberrant cell body conformations (asterisks) of the treated epimastigote parasites. (E, G) The normal morphology, including mitochondria (m), kinetoplasts (k), nucleus (N), and the entire plasma membrane, of trypomastigote parasites that were untreated. (F, H) The treated parasites showed blebs emerging from the cell body and flagella as well as membrane extensions. Adapted with permission from ref (5). Copyright 2013 Elsevier Ltd.

The antimicrobial peptide melittin can also effectively target the parasite *Trypanosoma cruzi*, which causes Chagas disease. The peptides lytic action is effective at various stages of the parasite including epimastigotes, *i.e.*, vector stage, trypomastigotes, *i.e.*, infectious non-proliferative stage, and the intracellular amastigote proliferative stage (Figure 5).^5^*T. cruzi* amastigotes are affected at melittin doses 100-fold less than the dose that is harmful to mammalian cells.^5^ It was further discovered that melittin triggers two distinct processes of death in the parasite, *i.e.*, autophagy and apoptosis.^5^

#### 4.1.3 Mellitin against *Entamoeba histolytica*

Few studies have demonstrated that the intestinal parasite *Entamoeba histolytica* is effectively killed by the hybrid form of the peptides melittin and cecropin (CM11).

*E. histolytica* was used to test CM11’s cytotoxicity with a coculture with Caco-2, a human colonic cancer cell line. When applied to *E. histolytica* trophozoites alone, the CM11 peptide demonstrated 93.7% antiparasitic efficacy at a concentration of 24 g/mL, whereas in the coculture condition the same peptide at the same concentration resulted in the death of 63.5% of the trophozoites. These results indicated that the parasite gained additional resistance against this peptide due to its cocultivation with host epithelial cells.^125^

### 4.2 Cecropin

Cecropins, identified in the hemolymph of *Hyalophora cecropia* (North America’s largest native moth), are antimicrobial peptides that constitute key effectors representing an unspecific or innate immunity component of insects.^126^ The number of amino acids in these linear cationic peptides varies greatly (31 and 37)^126^ among species. The cecropin family consists of five subgroups (A-−), together with other cecropin-like peptides called papiliocins, enbocins, sarcotoxions, spodopsins, and stomoxins,.^127,128^ An extensive range of antibacterial actions of cecropins has been demonstrated against both Gram-positive and Gram-negative bacteria and fungi. Cecropin A lyses bacteria, both Gram-positive and Gram-negative, by first attaching itself to the negatively charged membrane lipid via its highly positively charged N-terminus. Next, the hydrophobic C-terminus of cecropin A produces pore formation, which makes the membrane permeable and eventually kills the bacteria. HIV-1 replication has been found to be inhibited by cecropins and its derivatives, Shiva and SB-37.^129^ Studies has shown that cecropin A, which is isolated from *Hyalophora* and *Drosophila*, may impede the development of *Leishmania aethiopica*.^130^

### 4.3 Cathelicidins

The cathelicidins are another widely studied family of AMPs found in various species like pigs, cows, rabbits, and humans. Cationic amphiphilic peptides, cathelicidins have between 12–97 amino acids. Cathelicidin-derived peptides^131^ vary significantly and are present in a wide variety of structures with diverse functions. All of these members have a shared structure known as cathelin from which derivatives of cathelicidin are made through the process of proteolytic cleavage. This enzymatic process releases the mature COOH-terminal antimicrobial peptide.^132^ The only cathelicidin-type peptide that has been found in humans so far is cathelicidin antimicrobial peptide (CAMP), which is mostly found in macrophages, neutrophils, and other cells that are part of the host defensive response. Apart from its direct function against microbes, CAMP may also have indirect effects by controlling processes such as apoptosis, cell division, angiogenesis, cytokine release, inflammatory responses, and cell cycle arrest.

A prior study by Mark et al. identified that the cathelicidin-derived peptide LL-37 (Table 1), which is produced by cleaving the human cationic antimicrobial peptide-18 (hCAP-18) encoded by CAMP, was effective against leishmaniasis. Specifically, LL-37 could decrease the viability of *L. donovani* promastigotes by approximately 50% at the comparison to the untreated control. Moreover, *L. donovani* and *L. major* amastigotes were similarly sensitive to LL-37 peptide throughout their intramacrophage stage.^133^

Similarly, research was conducted on the bovine myeloid antimicrobial peptide (BMAP-28) on leishmaniasis. Isolated from bovine neutrophils, the BMAP-28 peptide is a cathelicidin-derived peptide with 28 amino acids. *Leishmania* promastigotes were studied for their *in vitro* activity against RI-BMAP-28, L-BMAP-28, and D-BMAP-28. Of these, the d-isoform exhibited the maximum efficacy in reducing promastigote viability. Additionally, it has been shown that BMAP-28 peptides are effective against amastigotes. Therefore, RI-BMAP-28, L-BMAP-28, and D-BMAP-28 may represent viable substitute therapies for leishmaniasis.^134^

### 4.4 Defensin

The initial classes of antimicrobial peptides discovered in mammals are defensins such as α, β, and θ and have a conserved six-cysteine signature with the other AMPs that have been reported so far.^135^ Defensins have a broad range of antimicrobial properties, including leishmanial, antiviral,^136,137^ antifungal,^138−140^ and antibacterial properties.^141^ It was discovered that the plant-derived defensin “‘*Vigna unguiculata* defensin” (Vu-Def) was efficient against *Leishmania amazonensis.* The γ-core domain—the primary domain linked to the peptide’s antimicrobial activity—was found by testing successively shorter versions of Vu-Def. Despite consisting solely of a few conserved amino acid residues, the discovered γ-core domain of Vu-Def maintained all of the peptide’s biological activity. This conserved area is essential to the peptide’s antibacterial properties. Additionally, it has been demonstrated that plant defensins, such as Vu-Def, are not harmful to mammalian cells, suggesting their potential as secure and efficient medicinal agents.^142^

### 4.5 Magainin

An African clawed frog (*Xenopus laevis*) produces a 23-residue peptide known as magainin.^143^ Magainins are a well-known class of α-helical peptides that function similarly to melittin.^144^ Following their attachment to negatively charged phospholipids, these antimicrobial peptides (AMPs) enter into cell membranes, causing cell lysis.^145,146^ Magainins are efficient against microorganisms and as antitumor agents^147,148^ without any toxicity to red blood cells.^149^

It has been discovered that certain magainins are efficient against *Leishmania* protozoans. *L. donovani* promastigote proliferation was reduced at micromolar concentrations by two hydrophobic magainin analogues, MG-H1 and MG-H2, as well as the original peptide F5W-magainin-2.^150^ Of these, MG-H2 exhibited the highest efficacy at micro molar concentration. These magainins cause a fast collapse of bioenergetics by breaking down parasite membranes. Recently, *Leishmania* promastigotes treated with the lysine-rich synthetic magainin analogue pexiganan have demonstrated its apoptotic effects. At concentrations between 10 μg/mL and 100 μg/mL, magainin-2, a vertebrate polycationic peptide, has cytotoxic effects on *Cryptosporidium parvum* sporozoites, causing a considerable reduction in their vitality to 9.7% after 60 min. On *C. parvum* oocysts, however, it is less effective; even after 180 min at 100 μg/mL, the viability is still above 65%. The thick wall of oocysts acts as a protective barrier, which may explain why the peptide’s alteration of the sporozoite’s apical complex prevents attachment and penetration of host cells.^151^

### 4.6 Dermaseptins

Amphibian skin naturally secretes dermaseptins, which are polycationic peptides that act as a barrier against microorganisms. Usually consisting of 27–34 amino acids and having a cationic amphipathic character, they have several analogues of amino acid sequence. When applied at extremely low dosages, dermaseptins can be fatal to several types of microorganisms such as yeast, fungi, bacteria, parasites, and enveloped viruses. Except for dermaseptin S4, which has strong hemolytic and antiprotozoan activity, none of the dermaseptins are harmful to mammalian cells.^152^

Additionally, it was discovered that the synthetic dermaseptin peptide (dermaseptin 01) was effective active against to *L. amazonensis* in the promastigote form^153^ at the concentration range of 1.0–256 μg/mL.

### 4.7 Eumenitin

In 2006, Konno et al. found eumenitin, a recently discovered antimicrobial peptide.^154^ They extracted eumenitin from the venom of the solitary wasp *Eumenes rubronotatus*. The wasp genus *Eumenes* belongs to the Eumeninae subfamily. With over 100 species and subspecies found all over the world, this species is a sizable and widely distributed genus. Eumenitin has a linear helical shape and consists of 15 amino acids (LNLKGIFKKVASLLT).^154^

This AMP has demonstrated efficacy against promastigotes of *L. major* promastigotes. Additionally, according to research by Rangel et al., peptides isolated from *Eumenes fraterculus* (eumenitin F; LNLKGLFKKVASLLT) and *Eumenes rubrofemoratus* (eumenitin R; LNLKGLIKKVASLLN) showed antileishmanial action against promastigotes of *L. major*.^155,156^

### 4.8 Histatin

Human oral antimicrobial peptides called histatins are linked to immunity and are produced into saliva by the salivary glands.^157^ There are 12 small histidine-rich cationic AMPs in the histatin family, among which histatin 1, 3, and 5 are the most prevalent.^158^

Histatins work well against a variety of microorganisms. Only Hst5, its d-enantiomer, and its synthetic analogue Dhvar4 have been examined on *L. donovani* for their antimicrobial activities. As studied by Ortega et al., *Leishmania* was susceptible to Hst5 at micromolar doses. Fatal concentrations of 14.4 μM for amastigotes and 7.3 μM for promastigotes was observed for Hst5. Further studies showed that compared to Hst-5, D-Hst5 and Dhvar4 were more effective on *L. donovani* and *L. pifanoi*.^159^

## 5 Antiviral Peptides

**Figure 6:**
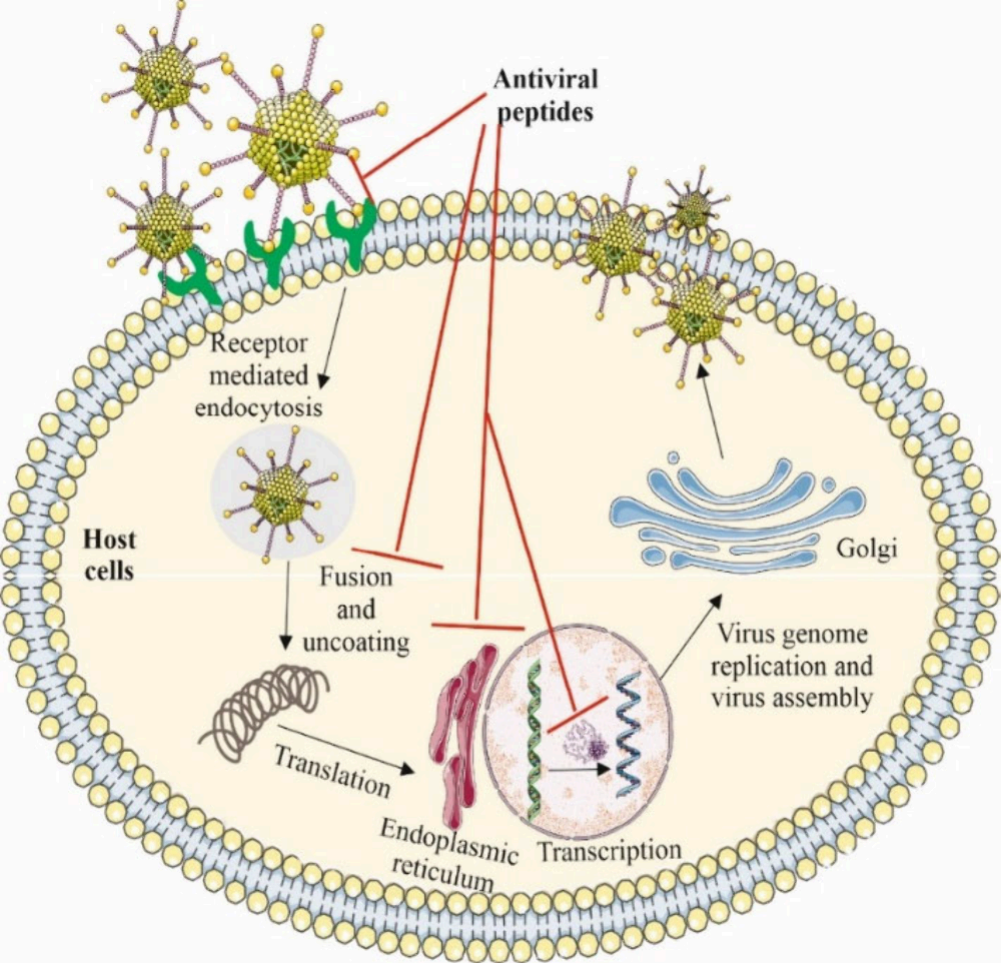
General mechanism of action of antiviral peptides to inhibit viral infections. The mechanism of antiviral peptides can alter nearly every stage of the viral life cycle; for example, they are able to suppress the receptor-mediated viral endocytosis, viral fusion, and uncoating in the host cell. Additionally, these peptides also inhibit the transcription and translation of the viral genome as well as the release of mature viral particles. Some parts of the figure were drawn by using pictures from Servier Medical Art. Servier Medical Art by Servier is licensed under a Creative Commons Attribution 4.0 Unported License https://creativecommons.org/licenses/by/4.0/.

Over the years, viral infections have made a substantial impact on global morbidity and mortality. Even with the presence of several therapeutic treatments for viral infections, the threat of new viruses having rapid replication and easy mutation, making them resistant, emphasizes the need for more potent treatments. AVPs have the ability to inhibit viral infections by targeting different phases of the viral life cycle like the membranes of enveloped viruses, preventing cellular penetration, limiting viral transcription and translation, and also preventing mature viral particles from budding (Figure 6).^160^

Here, some of the antiviral therapies based on peptides against some viral infection such as COVID-19, influenza, and dengue are discussed for their recent antiviral studies.

### 5.1 COVID-19

The pandemic COVID-19 driven by single stranded RNA SARS-CoV-2 virus has spurred international research toward efficient cures and preventative therapy. The use of peptide-based therapies is one exciting strategy that has drawn interest. Several approaches to using peptides to prevent SARS-CoV-2 entrance and replication have been investigated recently. The human spike protein facilitates envelope viral attachment to host cells through the action of human angiotensin-converting enzyme 2 (hACE2).^161^ It is possible to create peptides that specifically target the glycoprotein on the viral envelope to inhibit receptor attachment and fusion, which stops the virus from infecting host cells.

Zhang et al. have described the use of an engineered peptide-based inhibitor as an exclusive COVID-19 therapy. The^162^ S protein-binding portion of hACE2 has been divided into small fragments. Several hACE2 fragments with high affinity for binding SARS-CoV-2 exhibited strong antiviral activity. Zhang et al. developed a 23-mer peptide known as SBP1 using an automated fast-flow peptide synthesizer. The affinity between the SBP1 peptide and the SARS-CoV-2-RBD protein at the micromolar range was observed. The interest in using a peptide is heightened as an antiviral medication to treat COVID-19. The team also created an 85-mer N-terminal truncation of the hACE2 mini-protein, spike plug, to produce a stable and soluble S protein-binding mediator.^162^ Spike plug binds with the SARS-CoV-2-RBD protein with a nanomolecular affinity and possesses an α-helical shape. The association between the hACE2 amino acid residues and the S protein served as the purpose for the construction of the spike plug. Additionally, the spike plug has the ability to effectively prevent contact of the S protein with the hACE2 receptor, increasing the possibility that it may prevent interactions with the receptor.

Alternatively, SARS-CoV-2 fusion machinery can be targeted by the invention of coronavirus fusion inhibitors. A pan-coronavirus fusion inhibitor known as EK1 was developed to selectively target the HR1 domains of HCoV S proteins. It was demonstrated to work effectively in MERS (Middle East respiratory syndrome) as well.^163^ A IC_50_ value of 2.38 μM was observed for the EK1 molecule when tested on hACE2-expressing HEK293 transfected cells infected with the SARS-CoV-2 pseudovirus.^164^ In another work, Xia et al. produced a lipopeptide known as EK1C4 using a cholesterol molecule coupled to EK1. This lipopeptide had a very strong inhibitory effect on membrane fusion facilitated by the S protein of SARS-CoV-2. EK1C4 was shown to be 240× more effective than EK1 peptide in both *in vitro* and *in vivo* studies. EK1C4 has demonstrated encouraging outcomes for other coronaviruses, such as HCoVOC43 and MERS-CoV.^165^ Peptide inhibitors derived from HR1 and HR2, specifically named 2019-nCoV-HR1P and 2019-nCoV-HR2P, were developed for SARS-CoV-2 based on the findings from earlier inhibitors designed for SARS-CoV and MERS-CoV. These inhibitors targeted the S-heptad repeat 1 (HR1) region of the fusion machinery. The 2019-nCoV-HR2P inhibitor demonstrated significant fusion-blocking activity in laboratory tests, with an IC_50_ of 0.18 μM^164^.

Struck et al, demonstrated that a hexapeptide (YKYRYL) created utilizing a naturally occurring hexapeptide present in the SARS-CoV RBD decreased viral infection *in vitro*(166) in an experiment using epithelial cell lines. A study by Wang et al. suggests that the Paneth cells in the Lieberkühn crypts release a peptide “HD5” which has a strong affinity for ACE2 receptors.^167^ Through competitive binding to the ligand-binding domains of the ACE2 receptor in molecular dynamics simulations, it was found that HD5 forms numerous hydrogen bonds that may protect against SARS-CoV-2 by obstructing the binding sites that the virus utilizes.

It has been discovered that a serine protease known as TMPRSS2 helps with SARS-CoV-2 priming. A naturally occurring protein α1-antitrypsin prevents SARS-CoV-2 from entering cells by obstructing TMPRSS2. The ability of peptide mimetic inhibitors of TMPRSS2 (MI-432 and MI-1900) to shield human airway cells against SARS-CoV-2 infection was shown by Bestle et al.^168^

The spike’s ability to function and the virus’s ability to merge with the cell are both hindered when α1-antitrypsin suppresses TMPRSS2, which typically aids the virus in preparing its spike for entrance. The antiviral activity was seen to be enhanced when both TMPRSS2 inhibitors were combined with the furin inhibitor MI-1851. This suggests that peptides targeting distinct cleavage sites may have synergistic effects. A strong wide-ranging action of peptide P9R (NGAICWGPCPTAFRQIGNCGRFRVRCCRIR) was reported by Zhao et al. against enveloped viruses. This AVP inhibits endosomal acidification by minimizing protons inside the endosome as its mode of action. P9R was altered to have a larger positive charge, and shown to have more antiviral efficacy against SARS-CoV-2 than wild-type P9R. The wild-type P9R was tested as AVP earlier with a similar mechanism of action, *i.e.*, to attach to viral glycoproteins and stop endosomal acidification.^169^

### 5.2 Influenza

Influenza is an acute respiratory condition that causes significant global economic losses in addition to high rates of morbidity and fatality. Influenza viruses are mostly spread by respiratory droplets, such as aerosols, that are released when an infected person breathes, sneezes, coughs, speaks, or sings. Influenza A (IAV) and influenza B (IBV) are the two primary human influenza viruses that annually produce seasonal flu outbreaks. In order to overcome pre-existing immunity and obtain a competitive advantage, IAV and IBV develops surface protein mutations that provide novel antigenic variations^170^ Seasonal vaccinations and some antiviral medications are some limited treatments for combating influenza. The strains that are included in each year’s seasonal flu vaccination are chosen through meticulous research, which adds a tremendous amount of work to the vaccine’s production. Since resistance to antiviral drugs is increasing and they have unfavorable side effects, the use of these drugs is restricted. Additionally, due to the highly mutative nature of these viruses, new antigenic variants are constantly emerging, which necessitates the urgent development of novel antiviral therapeutic strategies. Among these strategies, “peptide-based therapies”, a recently developed area of treatment against influenza viruses, is being investigated and appears to be promising.^171^

#### 5.2.1 Peptides Binding Hemagglutinin

Together with neuraminidase (NA), hemagglutinin (HA) is one of the viral surface proteins, a member of the class I fusion protein family. Structure-wise, HA is a large homotrimeric mushroom-like protein. Group 1 and group 2 are the two evolutionary groupings into which the 18 hemagglutinin (HA) subtypes are classified. Proteases split each monomer into two chains (HA1 and HA2), converting it from single-chain precursor HA to a fusogenic state. The globular head of the virus consists of HA1, which allows the virus to enter the endosome. The sialic acid (SA) on host membrane glycoproteins is identified by the receptor binding site (RBS) located at the top of the globular head. One SA molecule can be bound by each monomer with low affinity, but the total affinity and stability are increased by several bindings.^172^ This mushroom-like protein’s stem is made up of the HA2 chain. The fusion peptide (1–15 aa) makes it highly conserved among HAs. The hydrophobic fusion peptide is revealed by conformational changes in HA2 that result in the creation of the prehairpin structure due to endosomal acidification.^173^

Development of anti-influenza A virus drugs may find success in targeting RBS and fusion peptides, which are important components in HA-mediated processes. Numerous peptides have been found to prevent viruses from entering the host cell. They can be categorized according to HA’s binding location and, consequently, the process that HA is preventing from happening. The protein’s conformational rearrangement and internalization process may be hampered by compounds that interact with other areas of HA or compete with sialic acid binding at the RBS.

#### 5.2.2 Peptides Binding Sialic Acid

Jones et al. discovered an entry blocker (EB) peptide of 20 amino acids, which was obtained through the fibroblast growth factor 4 (FGF-4) signal sequence after studying a collection of 5 cell-penetrating peptides. Using the hemagglutination inhibition (HI) test, it was shown that the peptide prevented the virus from interacting with the host cell and that it had micromolar action against many IAV strains (H1N1, H2N2, H3N2, H5N1, H5N9, and H7N3). However, the peptide’s low therapeutic potency of 22 restricted its use.^174^ In their follow-up study, they determined that the minimum essential peptide sequence that preserved the lead EB’s antiviral activity was made up of 13 amino acids (B10). Further, a novel peptide of 16 amino acid (B7) was developed with a micromolar activity on PR/8 (H1N1) virus-infected Madin–Darby canine kidney (MDCK) cells, and a higher selectivity index^175^ was observed.

Matsubara et al. used affinity selection to screen 15-mer peptides with both H1 and H3 HAs, belonging to groups 1 and 2 of the phylogenetic tree, to find broad-spectrum compounds. They further performed another second session of selection using surface plasmon resonance (SPR) analysis to identify peptides binding to HA. The most potent s2 peptide was fragmented and subjected to Ala-scan, yielding 5-mer peptides with an elevated ability to defend against IAV infection (ARLPR). To enhance the antiviral action, stearic acid was coupled to all eight of the active peptides. It was anticipated that N-stearoyl peptides would enhance their activity by assembling in supramolecular structures like micelles.^176^ In a plaque reduction assay, C18-peptides demonstrated efficient action against MDCK cells infected with H1N1 and H3N2 viral strains at low micromolar ranges.

Mammals release lactoferrin in their milk, saliva, and tears^176^ which is involved in innate immunity. Pietrantoni et al. showed that bovine lactoferrin (bLf) had anti-influenza action, preventing the virus-induced apoptosis in MDCK cells.^177^ These findings prompted the study to concentrate on the individual lobes that make up bLf. In hemagglutination inhibition (HI) tests, the N-lobe was unsuccessful, while the C-lobe maintained its bLf activity against several virus strains (H1N1, H3N2, H5N1, and H7N1). Sequencing after Western blot analysis revealed the connection between the HA fusion peptide and the bLf C-lobe. The three identified peptides AGDDQGLDKCVPNSKEK, NGESTADWAKN, and SKHSSLDCVLRP were put to the test and showed efficacy against the same viral strains as previously documented, albeit with increased activity at concentrations ranging from femtomolar to picomolar. Additionally, they showed no toxicity up to 25 μM concentration, with a selectivity index of 106–108.^171,178^ Expanding upon the very effective tetrapeptides SLDC and SKHS, scientists utilized an Ala scan methodology to synthesize and assess eight supplementary peptides. The most interesting of them was the tetrapeptide SAHS, which showed subnanomolar levels of broad-spectrum antiviral activity. Based on their HI activity, docking studies showed that these peptides bind to the receptor binding site (RBS) of hemagglutinins (HAs), and where they clash with sialic acid.^179^

#### 5.2.3 Peptides Binding Neuraminidase

Neuraminidase (NA), an antigenic glycoprotein that is attached to the influenza virion’s surface envelope, is essential to the virus’s ability to replicate. As a result, it is a great therapeutic target for reducing influenza infections. As reported by Amri et al.,^180^ cyclic peptides have been shown to suppress H1N1 NA. Similarly, mimosine tetrapeptide (M-FFY) was found to have strong NA-inhibitory action by Upadhyay et al.^181^ A novel natural peptide, PGEKGPSGEAGTAGPPGTPGPQGL, was discovered by Li et al. using hydrolysates of cod skin. With a *K*_i_ (dissociation constant) value of 0.29 mM, this peptide directly bound to free enzymes and showed NA inhibitory activity. In another study, Chen et al. discovered an octapeptide (P2) derived from the binding pocket of oseltamivir in neuraminidase among a collection of 20 peptides. When P2 suppressed influenza neuraminidase activity at a dosage of 4.25 M, it demonstrated nanomolar affinity (11 nM) for the enzyme, effectively shielding MDCK cells against viral infection and influenza virus-induced mortality. Notably, P2 decreased mortality and inflammation caused by the influenza virus in infected mice, suggesting that it may offer protection against the deadly influenza virus in vivo.^182^

#### 5.2.4 Peptide Binding Polymerase Domain

The ribonucleoprotein (RNP) complex, which consists of the nucleoprotein and a trimeric RNA polymerase made up of the proteins PB1, PB2, and PA, is another possible target of anti-influenza peptides.^183^ The 5′-end of the viral RNA is bound by this enzyme complex more firmly than the 3′-end.^184^

### 5.3 Dengue

Dengue is a serious hazard to world health, yearly producing 390 million illnesses and 25 000 fatalities. *Aedes* mosquitoes carry the dengue virus (DENV), which causes dengue illnesses.^185^

DENV-1 through DENV-4 are the four antigenically distinct serotypes of the virus. A initial infection with a long-term protection against that serotype is provided by DENV-1 for life and temporary protection from the other three serotypes for about six months.

Dengvaxia (CYD-TDV), the only approved dengue vaccine, is restricted as it is not highly effective against DENV-1 and DENV-2 strains and can induce severe dengue in those who have never had the virus before.^185^ The only proven therapy for dengue infections is supportive care, which includes replacing lost fluid and taking analgesics. There are currently no clinically licensed antivirals for this condition.^186^

The envelope protein domain 3 (ED3) of the DENV is the primary target for highly effective virus-neutralizing antibodies.^187^ Cui et al. assessed several peptides that were created using the DENV E protein’s domain III. They discovered that P4, a peptide that targets the β3 integrin, inhibits DENV-2 binding with an IC_50_ of 19.08 ± 2.52 μM, thereby disrupting viral entry and demonstrating its potential as an antiviral agent. They further found that another peptide “P7” exhibits an inhibitory effect opposed to DENV-2 with an IC_50_ value of 12.86 ± 5.96 μM. The group suggested that P4 and P7 peptides occupied the DENV binding site on the integrin receptor to prevent DENV-2 entrance into human endothelial cell lines (HUVECs).^188^ Another study from Hrobowski et al. evaluated the capacity of peptides made from DENV E protein sequences and suggested that DN59 was the most effective peptide, which showed more than 99% suppression of the formation of DENV-2 plaque at concentrations below 25 μM and also exhibited 100% inhibitory efficacy against DENV-2 at 20 μM.^189^

The DENV NS1 protein size is between 46 to 55 kDa, based on its glycosylation status. It possesses several oligomer forms, like the ER-resident, secreted, and membrane-anchored forms, and can be found in various cellular sites.^190^ Four peptides (peptides 3, 4, 10, and 11) were found by Songprakhon et al. to attach to the DENV NS1 protein and efficiently prevent DENV infections. These peptides are able to spontaneously attach with the DENV-2 NS1 protein because they have very high negative binding free energies. At 4 h after infection, all four peptides at 10 μM concentration caused a significant decline of DENV-2 virions (42–57%) (Figure 6).

### 5.4 Enterovirus A71

Enterovirus A71 (EV-A71) is the primary pathogen that causes mouth, hand, and foot disease, belonging to the enterovirus A species. EV-A71 is also responsible for causing severe neurological complications. Lin et al.’s study was the first to describe the antiviral activity of peptides against EV-A71 in rhabdomyosarcoma (RD) cells.^191^ Lactoferrin suppresses many different strains of EV-A71, probably by targeting the viral structural protein (VP1) as well as host cell receptors (glycosaminoglycans and heparan sulfate). It was essential to preincubate cells with lactoferrin to observe an antiviral effect, and its *in vitro* efficiency improved significantly with longer preincubations.^191^ Lactoferrin alone provided 30% protection compared to the control group of EV-A71-infected mice.

Much research has been done on several antimicrobial peptides found in bee venom, such as mast cell degranulating peptide, melittin, apamin, and adolapin. In the research identified by Uddin et al., the antiviral effectiveness of bee venom against EV-A71 was investigated through an *in vitro* study, which indicated a potent antiviral characteristics even at low doses of 2.0 μg/mL. Melittin, a 26 amino acid long peptide (GIGAVLKVLTTGLPALISWIKRKRQQ) has a direct virucidal impact on EV-A71 infection. When melittin and the virus were cocultured for 30 min, the virus’s cytopathic impact was significantly reduced after 24 h of infection. VP1 mRNA levels were found to be four-times lower than those of the untreated virus. Notably, melittin’s efficacy was highlighted by the fact that even at the concentration of 2.0 μg/mL melittin showed significant viral reduction, and a concentration of 4.36 μg/mL resulted in 50% mast cell death.^192^

Chen et al. discovered that at the time of picornavirus infections, specifically EV-A71, the level of the 45-amino acid peptide known as human β-defensin 3 (hBD3) was elevated. Recombinant hBD3 protein was applied to EV-A71 externally and intracellularly to examine its function in suppressing viral infection. It was observed that the virus was only suppressed in the case of extracellular hBD3 protein application, suggesting that hBD3 functions outside of cells to prevent the entrance of EV-A71 in the initial phases of infection, protecting the cells in the process.^193^

Tan et al. evaluated 95 synthetic 15-mer peptides covering all 297 amino acids of the EV-A71 VP1 protein. Four peptides, including SP40, SP45, SP81, and SP82, were demonstrated to significantly (>80%) reduce EVA71 infection in rhabdomyosarcoma cells *in vitro*. The amino acid residues 118–132 of peptide SP40 (VP1) had the strongest antiviral properties out of the 95 peptides screened. It was discovered that the SP40 peptide’s positively charged amino acid sequence is crucial for EV-A71 inhibition. Notably, it also came to light that arginine on position 3 significantly enhanced the biological activity of the SP40 peptide.

It has been discovered that by pretreating cells with the peptide for 1 h before infection, SP40 may suppress EV-A7 and effectively prevent the virus from attaching to or entering the cells.

It was earlier found that through suppressing heparan sulfate, antiheparan sulfate peptides G1 and G2 have been shown to diminish the herpes simplex virus infection, where an anchoring protein facilitates the attachment of viruses to host cells.^194^ Two peptides G1 and G2 were examined by Tan et al. and preincubated with rhabdomyosarcoma cells for 1 h prior to EV-A71 infection, then peptide G2 was capable of reducing the infection up to 76.5% at 1000 μg/mL, as shown by the plaque assay and qRT-PCR.^195^ However, due to its high effective concentration required to inhibit viruses, considerable alterations in peptide sequence are required.

## 6 Conclusion and Future Perspective

Peptides have emerged as a distinct and promising therapeutic class, playing a crucial role in the pharmaceutical industry across multiple domains. Their rapid growth is driven by their versatility and efficacy, expanding their applications in drug delivery, design, and manufacturing. With favorable properties such as adaptive pharmacokinetics, minimal toxicity, and high specificity, peptides serve as a bridge between small molecules and biologics. The increasing range of peptide-based treatments is fueling advancements in peptide discovery and optimization, particularly in immunotherapy, metabolic disorders, infectious diseases, and oncology.

Furthermore, the development of customized peptide-based therapies and antimicrobial peptides underscores their potential to address critical medical needs. While traditional challenges such as short half-life, low *in vivo* stability, membrane impermeability, and poor oral bioavailability have been significant limitations, advancements in nanotechnology and targeted drug delivery methods are overcoming these obstacles. Extensive research in peptide discovery, manufacturing, and optimization is enhancing their therapeutic potential. Innovations in peptide design are improving efficacy through sustained-release formulations, enhanced stability, and increased bioavailability while minimizing off-target effects.

These advancements hold great promise in emerging fields including peptide-based vaccines, antimicrobial peptides (AMPs) for combating multidrug-resistant infections, and personalized medicine. Peptides regulating appetite and metabolism may offer novel treatments for obesity, while others are being explored for chronic pain management and the treatment of neurological disorders. Targeted drug delivery approaches are also being developed to treat challenging conditions, such as brain tumors and drug-resistant cells.

Additionally, peptides are being investigated for treating cardiovascular diseases, gastrointestinal disorders, and infectious diseases, as well as for vaccine development. Numerous therapeutic peptides are presently undergoing preclinical and clinical research, and several of them have already made their way onto the global market. The integration of artificial intelligence, computational biology, and advanced screening technologies is expected to accelerate the discovery and optimization. With a promising future ahead, therapeutic peptides will continue to shape modern medicine, driving long-term advancements in the field of healthcare.

## Abbreviation

MUC-1: mucin 1
HER2: human epidermal growth factor receptor 2
HPV16 E7: human papillomavirus type 16 E7
DCs: dendritic cells
VEGFR1: vascular endothelial growth factor receptor 1
VEGFR2: vascular endothelial growth factor receptor 2
HLA: human leukocyte antigen
MAGE-3: melanoma-associated antigen 3
GnRH: gonadotropin-releasing hormone

## References

[ref1] Li H.; Gao J. a.; Zhao F.; Liu X.; Ma B. Bioactive peptides from edible mushrooms—the preparation, mechanisms, structure—activity relationships and prospects. Foods 2023, 12 (15), 2935 10.3390/foods12152935. 37569204 PMC10417677

[ref2] Nguyen T. T. M.; Yi E.-J.; Jin X.; Zheng Q.; Park S.-J.; Yi G.-S.; Yang S.-J.; Yi T.-H. Sustainable dynamic wrinkle efficacy: non-invasive peptides as the future of botox alternatives. Cosmetics 2024, 11 (4), 118 10.3390/cosmetics11040118.

[ref3] Jang A.; Jo C.; Kang K.-S.; Lee M. Antimicrobial and human cancer cell cytotoxic effect of synthetic angiotensin-converting enzyme (ACE) inhibitory peptides. Food Chem. 2008, 107 (1), 327–336. 10.1016/j.foodchem.2007.08.036.

[ref4] Leal J.; Dong T.; Taylor A.; Siegrist E.; Gao F.; Smyth H. D. C.; Ghosh D. Mucus-penetrating phage-displayed peptides for improved transport across a mucus-like model. International journal of pharmaceutics 2018, 553 (1–2), 57–64. 10.1016/j.ijpharm.2018.09.055. 30268850 PMC6448585

[ref5] Adade C. M.; Oliveira I. R. S.; Pais J. A. R.; Souto-Padrón T. Melittin peptide kills Trypanosoma cruzi parasites by inducing different cell death pathways. Toxicon 2013, 69, 227–239. 10.1016/j.toxicon.2013.03.011. 23562368

[ref6] Rogol A. D.; Laffel L. M.; Bode B.; Sperling M. A. Celebration of a century of insulin therapy in children with type 1 diabetes. Archives of Disease in Childhood 2023, 108 (1), 3–10. 10.1136/archdischild-2022-323975. 35725290 PMC9763182

[ref7] Mahlapuu M.; Håkansson J.; Ringstad L.; Björn C. Antimicrobial peptides: an emerging category of therapeutic agents. Front. Cell. Infect. Microbiol. 2016, 6, 194 10.3389/fcimb.2016.00194. 28083516 PMC5186781

[ref8] Amso Z.; Hayouka Z. Antimicrobial random peptide cocktails: A new approach to fight pathogenic bacteria. Chem. Commun. 2019, 55 (14), 2007–2014. 10.1039/C8CC09961H. 30688322

[ref9] Lembo D.; Donalisio M.; Civra A.; Argenziano M.; Cavalli R. Nanomedicine formulations for the delivery of antiviral drugs: a promising solution for the treatment of viral infections. Expert Opinion on Drug Delivery 2018, 15 (1), 93–114. 10.1080/17425247.2017.1360863. 28749739

[ref10] Kumar P.; Kizhakkedathu J. N.; Straus S. K. Antimicrobial peptides: diversity, mechanism of action and strategies to improve the activity and biocompatibility in vivo. Biomolecules 2018, 8 (1), 4 10.3390/biom8010004. 29351202 PMC5871973

[ref11] Essa R. Z.; Wu Y.-s.; Batumalaie K.; Sekar M.; Poh C.-l. Antiviral peptides against SARS-CoV-2: therapeutic targets, mechanistic antiviral activity, and efficient delivery. Pharmacological Reports 2022, 74 (6), 1166–1181. 10.1007/s43440-022-00432-6. 36401119 PMC9676828

[ref12] Oren Z.; Shai Y. Mode of action of linear amphipathic α-helical antimicrobial peptides. Peptide Science 1998, 47 (6), 451–463. 10.1002/(SICI)1097-0282(1998)47:6<451::AID-BIP4>3.0.CO;2-F. 10333737

[ref13] Shai Y. Mechanism of the binding, insertion and destabilization of phospholipid bilayer membranes by α-helical antimicrobial and cell non-selective membrane-lytic peptides. Biochimica et Biophysica Acta (BBA)-Biomembranes 1999, 1462 (1–2), 55–70. 10.1016/S0005-2736(99)00200-X. 10590302

[ref14] Matsuzaki K.; Murase O.; Fujii N.; Miyajima K. An antimicrobial peptide, magainin 2, induced rapid flip-flop of phospholipids coupled with pore formation and peptide translocation. Biochemistry 1996, 35 (35), 11361–11368. 10.1021/bi960016v. 8784191

[ref15] Sable R.; Parajuli P.; Jois S. Peptides, peptidomimetics, and polypeptides from marine sources: a wealth of natural sources for pharmaceutical applications. Marine drugs 2017, 15 (4), 124 10.3390/md15040124. 28441741 PMC5408270

[ref16] Wang Z.; Zhang X. Isolation and identification of anti-proliferative peptides from Spirulina platensis using three-step hydrolysis. Journal of the Science of Food and Agriculture 2017, 97 (3), 918–922. 10.1002/jsfa.7815. 27218227

[ref17] Lingeman R. G.; Hickey R. J.; Malkas L. H. Expression of a novel peptide derived from PCNA damages DNA and reverses cisplatin resistance. Cancer chemotherapy and pharmacology 2014, 74, 981–993. 10.1007/s00280-014-2574-x. 25190177 PMC5458613

[ref18] Smith S. J.; Gu L.; Phipps E. A.; Dobrolecki L. E.; Mabrey K. S.; Gulley P.; Dillehay K. L.; Dong Z.; Fields G. B.; Chen Y.-R.; et al. A Peptide mimicking a region in proliferating cell nuclear antigen specific to key protein interactions is cytotoxic to breast cancer. Mol. Pharm. 2015, 87 (2), 263–276. 10.1124/mol.114.093211. PMC4293449 25480843

[ref19] Gu L.; Smith S.; Li C.; Hickey R. J.; Stark J. M.; Fields G. B.; Lang W. H.; Sandoval J. A.; Malkas L. H. A PCNA-derived cell permeable peptide selectively inhibits neuroblastoma cell growth. PloS one 2014, 9 (4), e94773 10.1371/journal.pone.0094773. 24728180 PMC3984256

[ref20] Chen Y.; Pullambhatla M.; Banerjee S. R.; Byun Y.; Stathis M.; Rojas C.; Slusher B. S.; Mease R. C.; Pomper M. G. Synthesis and biological evaluation of low molecular weight fluorescent imaging agents for the prostate-specific membrane antigen. Bioconjugate Chem. 2012, 23 (12), 2377–2385. 10.1021/bc3003919. PMC4131203 23157641

[ref21] Yamamoto S.; Nishimura K.; Morita K.; Kanemitsu S.; Nishida Y.; Morimoto T.; Aoi T.; Tamura A.; Maruyama T. Microenvironment pH-induced selective cell death for potential cancer therapy using nanofibrous self-assembly of a peptide amphiphile. Biomacromolecules 2021, 22 (6), 2524–2531. 10.1021/acs.biomac.1c00267. 33960189

[ref22] Ma L.; Huang S.; Xie H.; Ma P.; Jia B.; Yao Y.; Gao Y.; Li W.; Song J.; Zhang W. Influence of chain length on the anticancer activity of the antimicrobial peptide CAMEL with fatty acid modification. Eur. J. Med. Chem. 2022, 239, 114557 10.1016/j.ejmech.2022.114557. 35759906

[ref23] Feng Z.; Wang H.; Chen X.; Xu B. Self-assembling ability determines the activity of enzyme-instructed self-assembly for inhibiting cancer cells. J. Am. Chem. Soc. 2017, 139 (43), 15377–15384. 10.1021/jacs.7b07147. 28990765 PMC5669277

[ref24] van de Kamp M.; Silvestrini M. C.; Brunori M.; van Beeumen J.; Hali F. C.; Canters G. W. Involvement of the hydrophobic patch of azurin in the electron-transfer reactions with cytochrome c551 and nitrite reductase. European journal of biochemistry 1990, 194 (1), 109–118. 10.1111/j.1432-1033.1990.tb19434.x. 2174771

[ref25] Punj V.; Bhattacharyya S.; Saint-Dic D.; Vasu C.; Cunningham E. A.; Graves J.; Yamada T.; Constantinou A. I.; Christov K.; White B.; et al. Bacterial cupredoxin azurin as an inducer of apoptosis and regression in human breast cancer. Oncogene 2004, 23 (13), 2367–2378. 10.1038/sj.onc.1207376. 14981543

[ref26] Yamada T.; Goto M.; Punj V.; Zaborina O.; Chen M. L.; Kimbara K.; Majumdar D.; Cunningham E.; Das Gupta T. K.; Chakrabarty A. M. Bacterial redox protein azurin, tumor suppressor protein p53, and regression of cancer. Proc. Natl. Acad. Sci. U. S. A. 2002, 99 (22), 14098–14103. 10.1073/pnas.222539699. 12393814 PMC137843

[ref27] Yamada T.; Mehta R. R.; Lekmine F.; Christov K.; King M. L.; Majumdar D.; Shilkaitis A.; Green A.; Bratescu L.; Beattie C. W.; et al. A peptide fragment of azurin induces a p53-mediated cell cycle arrest in human breast cancer cells. Mol. Cancer Ther. 2009, 8 (10), 2947–2958. 10.1158/1535-7163.MCT-09-0444. 19808975

[ref28] Ramachandran S.; Mandal M. Induction of apoptosis of azurin synthesized from P. áaeruginosa MTCC 2453 against Dalton’s lymphoma ascites model. Biomedicine & pharmacotherapy 2011, 65 (7), 461–466. 10.1016/j.biopha.2011.03.006. 22000294

[ref29] Zhang Y.; Zhang Y.; Xia L.; Zhang X.; Ding X.; Yan F.; Wu F. Escherichia coli Nissle 1917 targets and restrains mouse B16 melanoma and 4T1 breast tumors through expression of azurin protein. Applied and environmental microbiology 2012, 78 (21), 7603–7610. 10.1128/AEM.01390-12. 22923405 PMC3485711

[ref30] Soleimani M.; Sadeghi H. M.; Jahanian-Najafabadi A. A Bi-functional targeted P28-NRC chimeric protein with enhanced cytotoxic effects on breast cancer cell lines. Iran. J. Pharm. Res. 2019, 18 (2), 735 10.22037/ijpr.2019.2392. 31531057 PMC6706745

[ref31] Noei A.; Nili-Ahmadabadi A.; Soleimani M. The enhanced cytotoxic effects of the p28-apoptin chimeric protein as a novel anti-cancer agent on breast cancer cell lines. Drug Research 2019, 69 (03), 144–150. 10.1055/a-0654-4952. 30060264

[ref32] Shahbazi S.; Bolhassani A. Comparison of six cell penetrating peptides with different properties for in vitro and in vivo delivery of HPV16 E7 antigen in therapeutic vaccines. International Immunopharmacology 2018, 62, 170–180. 10.1016/j.intimp.2018.07.006. 30015237

[ref33] Chatzisideri T.; Leonidis G.; Sarli V. Cancer-targeted delivery systems based on peptides. Future Medicinal Chemistry 2018, 10 (18), 2201–2226. 10.4155/fmc-2018-0174. 30043641

[ref34] Raucher D.; Ryu J. S. Cell-penetrating peptides: strategies for anticancer treatment. Trends in molecular medicine 2015, 21 (9), 560–570. 10.1016/j.molmed.2015.06.005. 26186888

[ref35] Bernardes N.; Abreu S.; Carvalho F. A.; Fernandes F.; Santos N. C.; Fialho A. M. Modulation of membrane properties of lung cancer cells by azurin enhances the sensitivity to EGFR-targeted therapy and decreased β1 integrin-mediated adhesion. Cell Cycle 2016, 15 (11), 1415–1424. 10.1080/15384101.2016.1172147. 27096894 PMC4934055

[ref36] Bernardes N.; Garizo A. R.; Pinto S. N.; Caniço B.; Perdigão C.; Fernandes F.; Fialho A. M. Azurin interaction with the lipid raft components ganglioside GM-1 and caveolin-1 increases membrane fluidity and sensitivity to anti-cancer drugs. Cell Cycle 2018, 17 (13), 1649–1666. 10.1080/15384101.2018.1489178. 29963969 PMC6133334

[ref37] Wu Y. C.; Liu X.; Wang J. L.; Chen X. L.; Lei L.; Han J.; Jiang Y. S.; Ling Z. Q. Soft-shelled turtle peptide modulates microRNA profile in human gastric cancer AGS cells. Oncol. Lett. 2018, 15 (3), 3109–3120. 10.3892/ol.2017.7692. 29435044 PMC5778892

[ref38] Hou J.; Deng M.; Li X.; Liu W.; Chu X.; Wang J.; Chen F.; Meng S. Chaperone gp96 mediates ER-α36 cell membrane expression. Oncotarget 2015, 6 (31), 31857 10.18632/oncotarget.5273. 26396174 PMC4741645

[ref39] Li X.; Wang B.; Liu W.; Gui M.; Peng Z.; Meng S. Blockage of conformational changes of heat shock protein gp96 on cell membrane by a α-helix peptide inhibits HER2 dimerization and signaling in breast cancer. PLoS One 2015, 10 (4), e0124647 10.1371/journal.pone.0124647. 25898135 PMC4405268

[ref40] Qian L.; Fan H.; Ju Y.; Chen L.; Li X.; Ye X.; Luo Y.; Li C.; Meng S. A peptide-based inhibitor of gp96 suppresses HBsAg expression and HBV replication by upregulation of p53. Journal of General Virology 2019, 100 (8), 1241–1252. 10.1099/jgv.0.001289. 31204972

[ref41] Rubinstein D. B.; Stortchevoi A.; Boosalis M.; Ashfaq R.; Ghebrehiwet B.; Peerschke E. I. B.; Calvo F.; Guillaume T. Receptor for the globular heads of C1q (gC1q-R, p33, hyaluronan-binding protein) is preferentially expressed by adenocarcinoma cells. International journal of cancer 2004, 110 (5), 741–750. 10.1002/ijc.20105. 15146564

[ref42] Simón-Gracia L.; Hunt H.; Teesalu T. Peritoneal carcinomatosis targeting with tumor homing peptides. Molecules 2018, 23 (5), 1190 10.3390/molecules23051190. 29772690 PMC6100015

[ref43] d’Avanzo N.; Torrieri G.; Figueiredo P.; Celia C.; Paolino D.; Correia A.; Moslova K.; Teesalu T.; Fresta M.; Santos H. A. LinTT1 peptide-functionalized liposomes for targeted breast cancer therapy. International journal of pharmaceutics 2021, 597, 120346 10.1016/j.ijpharm.2021.120346. 33545283

[ref44] Morris S.; Ahmad N.; André S.; Kaltner H.; Gabius H.-J.; Brenowitz M.; Brewer F. Quaternary solution structures of galectins-1,-3, and-7. Glycobiology 2004, 14 (3), 293–300. 10.1093/glycob/cwh029. 14693909

[ref45] Kumar S. R.; Deutscher S. L. 111In-labeled galectin-3–targeting peptide as a SPECT agent for imaging breast tumors. J. Nucl. Med. 2008, 49 (5), 796–803. 10.2967/jnumed.107.048751. 18413389

[ref46] Zamani P.; Navashenaq J. G.; Teymouri M.; Karimi M.; Mashreghi M.; Jaafari M. R. Combination therapy with liposomal doxorubicin and liposomal vaccine containing E75, an HER-2/neu-derived peptide, reduces myeloid-derived suppressor cells and improved tumor therapy. Life sciences 2020, 252, 117646 10.1016/j.lfs.2020.117646. 32272178

[ref47] Shariat S.; Badiee A.; Jalali S. A.; Mansourian M.; Yazdani M.; Mortazavi S. A.; Jaafari M. R. P5 HER2/neu-derived peptide conjugated to liposomes containing MPL adjuvant as an effective prophylactic vaccine formulation for breast cancer. Cancer letters 2014, 355 (1), 54–60. 10.1016/j.canlet.2014.09.016. 25224570

[ref48] Gholizadeh Z.; Tavakkol-Afshari J.; Nikpoor A. R.; Jalali S. A.; Jaafari M. R. Enhanced immune response induced by P5 HER2/neu-derived peptide-pulsed dendritic cells as a preventive cancer vaccine. Journal of Cellular and Molecular Medicine 2018, 22 (1), 558–567. 10.1111/jcmm.13343. 28944998 PMC5742681

[ref49] Jalali S. A.; Sankian M.; Tavakkol-Afshari J.; Jaafari M. R. Induction of tumor-specific immunity by multi-epitope rat HER2/neu-derived peptides encapsulated in LPD Nanoparticles. Nanomedicine: Nanotechnology, Biology and Medicine 2012, 8 (5), 692–701. 10.1016/j.nano.2011.09.010. 22024191

[ref50] Mansourian M.; Badiee A.; Jalali S. A.; Shariat S.; Yazdani M.; Amin M.; Jaafari M. R. Effective induction of anti-tumor immunity using p5 HER-2/neu derived peptide encapsulated in fusogenic DOTAP cationic liposomes co-administrated with CpG-ODN. Immunology letters 2014, 162 (1), 87–93. 10.1016/j.imlet.2014.07.008. 25086399

[ref51] Vacchelli E.; Galluzzi L.; Eggermont A.; Fridman W. H.; Galon J.; Sautès-Fridman C.; Tartour E.; Zitvogel L.; Kroemer G. Trial watch: FDA-approved Toll-like receptor agonists for cancer therapy. Oncoimmunology 2012, 1 (6), 894–907. 10.4161/onci.20931. 23162757 PMC3489745

[ref52] Casella C. R.; Mitchell T. C. Putting endotoxin to work for us: monophosphoryl lipid A as a safe and effective vaccine adjuvant. Cellular and molecular life sciences 2008, 65, 3231–3240. 10.1007/s00018-008-8228-6. 18668203 PMC2647720

[ref53] Zamani P.; Navashenaq J. G.; Nikpoor A. R.; Hatamipour M.; Oskuee R. K.; Badiee A.; Jaafari M. R. MPL nano-liposomal vaccine containing P5 HER2/neu-derived peptide pulsed PADRE as an effective vaccine in a mice TUBO model of breast cancer. J. Controlled Release 2019, 303, 223–236. 10.1016/j.jconrel.2019.04.019. 30999007

[ref54] Xin H.; Jiang X.; Gu J.; Sha X.; Chen L.; Law K.; Chen Y.; Wang X.; Jiang Y.; Fang X. Angiopep-conjugated poly (ethylene glycol)-co-poly (ε-caprolactone) nanoparticles as dual-targeting drug delivery system for brain glioma. Biomaterials 2011, 32 (18), 4293–4305. 10.1016/j.biomaterials.2011.02.044. 21427009

[ref55] Xin H.; Sha X.; Jiang X.; Zhang W.; Chen L.; Fang X. Anti-glioblastoma efficacy and safety of paclitaxel-loading Angiopep-conjugated dual targeting PEG-PCL nanoparticles. Biomaterials 2012, 33 (32), 8167–8176. 10.1016/j.biomaterials.2012.07.046. 22889488

[ref56] Soroceanu L.; Gillespie Y.; Khazaeli M. B.; Sontheimer H. Use of chlorotoxin for targeting of primary brain tumors. Cancer Res. 1998, 58 (21), 4871–4879. 9809993

[ref57] Sattiraju A.; Sai K. K. S.; Xuan A.; Pandya D. N.; Almaguel F. G.; Wadas T. J.; Herpai D. M.; Debinski W.; Mintz A. IL13RA2 targeted alpha particle therapy against glioblastomas. Oncotarget 2017, 8 (26), 42997 10.18632/oncotarget.17792. 28562337 PMC5522122

[ref58] Wang B.; Lv L.; Wang Z.; Jiang Y.; Lv W.; Liu X.; Wang Z.; Zhao Y.; Xin H.; Xu Q. Improved anti-glioblastoma efficacy by IL-13Rα2 mediated copolymer nanoparticles loaded with paclitaxel. Sci. Rep 2015, 5, 16589 10.1038/srep16589. 26567528 PMC4645113

[ref59] Shoshan-Barmatz V.; De Pinto V.; Zweckstetter M.; Raviv Z.; Keinan N.; Arbel N. VDAC, a multi-functional mitochondrial protein regulating cell life and death. Molecular aspects of medicine 2010, 31 (3), 227–285. 10.1016/j.mam.2010.03.002. 20346371

[ref60] Shteinfer-Kuzmine A.; Arif T.; Krelin Y.; Tripathi S. S.; Paul A.; Shoshan-Barmatz V. Mitochondrial VDAC1-based peptides: Attacking oncogenic properties in glioblastoma. Oncotarget 2017, 8 (19), 31329 10.18632/oncotarget.15455. 28412744 PMC5458211

[ref61] Friedmann-Morvinski D.; Narasimamurthy R.; Xia Y.; Myskiw C.; Soda Y.; Verma I. M. Targeting NF-κB in glioblastoma: A therapeutic approach. Sci. Adv. 2016, 2 (1), e1501292 10.1126/sciadv.1501292. 26824076 PMC4730860

[ref62] Han L.; Zhang A.; Wang H.; Pu P.; Jiang X.; Kang C.; Chang J. Tat-BMPs-PAMAM conjugates enhance therapeutic effect of small interference RNA on U251 glioma cells in vitro and in vivo. Human gene therapy 2010, 21 (4), 417–426. 10.1089/hum.2009.087. 19899955

[ref63] Gupta B.; Levchenko T. S.; Torchilin V. P. TAT peptide-modified liposomes provide enhanced gene delivery to intracranial human brain tumor xenografts in nude mice. Oncology research 2006, 16 (8), 351–359. 10.3727/000000006783980946. 17913043

[ref64] Rousselle C.; Clair P.; Lefauconnier J.-M.; Kaczorek M.; Scherrmann J.-M.; Temsamani J. New advances in the transport of doxorubicin through the blood-brain barrier by a peptide vector-mediated strategy. Mol. Pharmacol. 2000, 57 (4), 679–686. 10.1016/S0026-895X(24)26466-X. 10727512

[ref65] Rousselle C.; Clair P.; Temsamani J.; Scherrmann J.-M. Improved brain delivery of benzylpenicillin with a peptide-vector-mediated strategy. J. Drug Targeting 2002, 10 (4), 309–315. 10.1080/10611860290031886. 12164379

[ref66] Kraan C. M.; Godler D. E.; Amor D. J. Epigenetics of fragile X syndrome and fragile X-related disorders. Developmental Medicine & Child Neurology 2019, 61 (2), 121–127. 10.1111/dmcn.13985. 30084485

[ref67] Wyck S.; Herrera C.; Requena C. E.; Bittner L.; Hajkova P.; Bollwein H.; Santoro R. Oxidative stress in sperm affects the epigenetic reprogramming in early embryonic development. Epigenetics Chromatin 2018, 11, 60 10.1186/s13072-018-0224-y. 30333056 PMC6192351

[ref68] Pan W.; Yu H.; Zheng B.; Gao Y.; Li P.; Huang Q.; Xie C.; Ge X. Upregulation of MiR-369–3p suppresses cell migration and proliferation by targeting SOX4 in Hirschsprung’s disease. Journal of pediatric surgery 2017, 52 (8), 1363–1370. 10.1016/j.jpedsurg.2017.04.002. 28412032

[ref69] Wang G.; Zhang L.; Wang H.; Cui M.; Liu W.; Liu Y.; Wu X. Demethylation of GFRA4 promotes cell proliferation and invasion in Hirschsprung disease. DNA and Cell Biology 2018, 37 (4), 316–324. 10.1089/dna.2017.3928. 29634418

[ref70] Glahn A.; Riera Knorrenschild R.; Rhein M.; Haschemi Nassab M.; Gröschl M.; Heberlein A.; Muschler M.; Frieling H.; Bleich S.; Hillemacher T. Alcohol-induced changes in methylation status of individual CpG sites, and serum levels of vasopressin and atrial natriuretic peptide in alcohol-dependent patients during detoxification treatment. European addiction research 2014, 20 (3), 143–150. 10.1159/000357473. 24356727

[ref71] Hillemacher T.; Frieling H.; Luber K.; Yazici A.; Muschler M. A. N.; Lenz B.; Wilhelm J.; Kornhuber J.; Bleich S. Epigenetic regulation and gene expression of vasopressin and atrial natriuretic peptide in alcohol withdrawal. Psychoneuroendocrinology 2009, 34 (4), 555–560. 10.1016/j.psyneuen.2008.10.019. 19046820

[ref72] Yedery R. D.; Jerse A. E. Augmentation of cationic antimicrobial peptide production with histone deacetylase inhibitors as a novel epigenetic therapy for bacterial infections. Antibiotics 2015, 4 (1), 44–61. 10.3390/antibiotics4010044. 27025614 PMC4790325

[ref73] Philippeit C.; Busch M.; Dünker N. Epigenetic control of trefoil factor family (TFF) peptide expression in human retinoblastoma cell lines. Cellular Physiology and Biochemistry 2014, 34 (3), 1001–1014. 10.1159/000366316. 25199519

[ref74] Brocks D.; Schmidt C. R.; Daskalakis M.; Jang H. S.; Shah N. M.; Li D.; Li J.; Zhang B.; Hou Y.; Laudato S.; et al. DNMT and HDAC inhibitors induce cryptic transcription start sites encoded in long terminal repeats. Nat. Genet. 2017, 49 (7), 1052–1060. 10.1038/ng.3889. 28604729 PMC6005702

[ref75] Licht J. D. DNA methylation inhibitors in cancer therapy: the immunity dimension. Cell 2015, 162 (5), 938–939. 10.1016/j.cell.2015.08.005. 26317460

[ref76] Alonso-Álvarez S.; Pardal E.; Sánchez-Nieto D.; Navarro M.; Caballero M. D.; Mateos M. V.; Martín A. Plitidepsin: design, development, and potential place in therapy. Drug Design, Development and Therapy 2017, Volume11, 253–264. 10.2147/DDDT.S94165. PMC5261604 28176904

[ref77] Qi Y.; Wang D.; Wang D.; Jin T.; Yang L.; Wu H.; Li Y.; Zhao J.; Du F.; Song M.; et al. HEDD: the human epigenetic drug database. Database 2016, 2016, baw159 10.1093/database/baw159. 28025347 PMC5199199

[ref78] Chiappinelli K. B.; Strissel P. L.; Desrichard A.; Li H.; Henke C.; Akman B.; Hein A.; Rote N. S.; Cope L. M.; Snyder A.; et al. Inhibiting DNA methylation causes an interferon response in cancer via dsRNA including endogenous retroviruses. Cell 2015, 162 (5), 974–986. 10.1016/j.cell.2015.07.011. 26317466 PMC4556003

[ref79] Khavinson V. K.; Solov’ev A. Y.; Tarnovskaya S. I.; Lin’kova N. S. Mechanism of biological activity of short peptides: cell penetration and epigenetic regulation of gene expression. Biology Bulletin Reviews 2013, 3, 451–455. 10.1134/S2079086413060042.

[ref80] Khavinson V. K.; Solov’ev A. I.; Zhilinskiĭ D. V.; Shataeva L. K.; Vanyushin B. F. Epigenetic aspects of peptide regulation of aging. Adv. Gerontol. 2012, 2, 277–286. 10.1134/S2079057012040091. 22708439

[ref81] Ashapkin V. V.; Linkova N. S.; Khavinson V. K.; Vanyushin B. F. Epigenetic mechanisms of peptidergic regulation of gene expression during aging of human cells. Biochemistry (Moscow) 2015, 80, 310–322. 10.1134/S0006297915030062. 25761685

[ref82] Lin’Kova N. S.; Drobintseva A. O.; Orlova O. A.; Kuznetsova E. P.; Polyakova V. O.; Kvetnoy I. M.; Khavinson V. K. Peptide regulation of skin fibroblast functions during their aging in vitro. Bull. Exp. Biol. Med. 2016, 161 (1), 175–178. 10.1007/s10517-016-3370-x. 27259496

[ref83] Wan X.; Liu H.; Sun Y.; Zhang J.; Chen X.; Chen N. Lunasin: A promising polypeptide for the prevention and treatment of cancer. Oncology letters 2017, 13 (6), 3997–4001. 10.3892/ol.2017.6017. 28599405 PMC5453050

[ref84] Liu J.; Jia S. H.; Kirberger M.; Chen N. Lunasin as a promising health-beneficial peptide. Eur. Rev. Med. Pharmacol. Sci. 2014, 18 (14), 2070–2075. 25027349

[ref85] Sarkar A.; Gogia N.; Glenn N.; Singh A.; Jones G.; Powers N.; Srivastava A.; Kango-Singh M.; Singh A. A soy protein Lunasin can ameliorate amyloid-beta 42 mediated neurodegeneration in Drosophila eye. Sci. Rep 2018, 8, 13545 10.1038/s41598-018-31787-7. 30202077 PMC6131139

[ref86] Du L.; Risinger A. L.; King J. B.; Powell D. R.; Cichewicz R. H. A potent HDAC inhibitor, 1-alaninechlamydocin, from a Tolypocladium sp. induces G2/M cell cycle arrest and apoptosis in MIA PaCa-2 cells. J. Nat. Prod. 2014, 77 (7), 1753–1757. 10.1021/np500387h. 24999749 PMC4113265

[ref87] De Schepper S.; Bruwiere H.; Verhulst T.; Steller U.; Andries L.; Wouters W.; Janicot M.; Arts J.; Van heusden J. Inhibition of histone deacetylases by chlamydocin induces apoptosis and proteasome-mediated degradation of survivin. J. Pharmacol. Exp. Ther. 2003, 304 (2), 881–888. 10.1124/jpet.102.042903. 12538846

[ref88] Kumarasinghe I. R.; Woster P. M. Cyclic peptide inhibitors of lysine-specific demethylase 1 with improved potency identified by alanine scanning mutagenesis. European journal of medicinal chemistry 2018, 148, 210–220. 10.1016/j.ejmech.2018.01.098. 29459279 PMC5837957

[ref89] Forneris F.; Binda C.; Adamo A.; Battaglioli E.; Mattevi A. Structural basis of LSD1-CoREST selectivity in histone H3 recognition. J. Biol. Chem. 2007, 282 (28), 20070–20074. 10.1074/jbc.C700100200. 17537733

[ref90] Shortridge M. D.; Walker M. J.; Pavelitz T.; Chen Y.; Yang W.; Varani G. A macrocyclic peptide ligand binds the oncogenic MicroRNA-21 precursor and suppresses dicer processing. ACS Chem. Biol. 2017, 12 (6), 1611–1620. 10.1021/acschembio.7b00180. 28437065 PMC5512579

[ref91] Zhang X.; Sha M.; Yao Y.; Da J.; Jing D. Increased B-type-natriuretic peptide promotes myocardial cell apoptosis via the B-type-natriuretic peptide/long non-coding RNA LSINCT5/caspase-1/interleukin 1β signaling pathway. Molecular Medicine Reports 2015, 12 (5), 6761–6767. 10.3892/mmr.2015.4247. 26323562 PMC4626192

[ref92] Garay H.; Espinosa L. A.; Perera Y.; Sánchez A.; Diago D.; Perea S. E.; Besada V.; Reyes O.; González L. J. Characterization of low-abundance species in the active pharmaceutical ingredient of CIGB-300: A clinical-grade anticancer synthetic peptide. J. Peptide Sci. 2018, 24 (6), e3081 10.1002/psc.3081. 29676523

[ref93] Perea S. E.; Reyes O.; Baladron I.; Perera Y.; Farina H.; Gil J.; Rodriguez A.; Bacardi D.; Marcelo J. L.; Cosme K.; et al. CIGB-300, a novel proapoptotic peptide that impairs the CK2 phosphorylation and exhibits anticancer properties both in vitro and in vivo. Mol. Cell. Biochem. 2008, 316, 163–167. 10.1007/s11010-008-9814-5. 18575815

[ref94] Rodríguez-Ulloa A.; Ramos Y.; Gil J.; Perera Y.; Castellanos-Serra L.; García Y.; Betancourt L.; Besada V.; González L. J.; Fernández-de-Cossio J.; et al. Proteomic profile regulated by the anticancer peptide CIGB-300 in non-small cell lung cancer (NSCLC) cells. J. Proteome Res. 2010, 9 (10), 5473–5483. 10.1021/pr100728v. 20804217

[ref95] Bernhardt S. L.; Gjertsen M. K.; Trachsel S.; Møller M.; Eriksen J. A.; Meo M.; Buanes T.; Gaudernack G. Telomerase peptide vaccination of patients with non-resectable pancreatic cancer: a dose escalating phase I/II study. British journal of cancer 2006, 95 (11), 1474–1482. 10.1038/sj.bjc.6603437. 17060934 PMC2360729

[ref96] Chiangjong W.; Chutipongtanate S.; Hongeng S. Anticancer peptide: Physicochemical property, functional aspect and trend in clinical application. International journal of oncology 2020, 57 (3), 678–696. 10.3892/ijo.2020.5099. 32705178 PMC7384845

[ref97] Lubamba B.; Dhooghe B.; Noel S.; Leal T. Cystic fibrosis: insight into CFTR pathophysiology and pharmacotherapy. Clinical biochemistry 2012, 45 (15), 1132–1144. 10.1016/j.clinbiochem.2012.05.034. 22698459

[ref98] Fellner R. C.; Terryah S. T.; Tarran R. Inhaled protein/peptide-based therapies for respiratory disease. Mol. cell. Pediatr. 2016, 3, 16 10.1186/s40348-016-0044-8. 27098663 PMC4839019

[ref99] Sala V.; Murabito A.; Ghigo A. Inhaled biologicals for the treatment of cystic fibrosis. Recent Patents on Inflammation & Allergy Drug Discovery 2019, 13 (1), 19–26. 10.2174/1872213X12666181012101444. 30318010 PMC6751348

[ref100] Bhagirath A. Y.; Li Y.; Somayajula D.; Dadashi M.; Badr S.; Duan K. Cystic fibrosis lung environment and Pseudomonas aeruginosa infection. BMC Pulm. Med. 2016, 16, 174 10.1186/s12890-016-0339-5. 27919253 PMC5139081

[ref101] Ong V.; Mei V.; Cao L.; Lee K.; Chung E. J. Nanomedicine for cystic fibrosis. SLAS TECHNOLOGY: Translating Life Sciences Innovation 2019, 24 (2), 169–180. 10.1177/2472630318824334. 30707858

[ref102] Savla R.; Minko T. Nanotechnology approaches for inhalation treatment of fibrosis. J. Drug Targeting 2013, 21 (10), 914–925. 10.3109/1061186X.2013.829078. PMC4219586 23978292

[ref103] Yan Z.; Wang D.; An C.; Xu H.; Zhao Q.; Shi Y.; Song N.; Deng B.; Guo X.; Rao J.; et al. The antimicrobial peptide YD attenuates inflammation via miR-155 targeting CASP12 during liver fibrosis. Acta Pharm. Sin. B 2021, 11 (1), 100–111. 10.1016/j.apsb.2020.07.004. 33532183 PMC7838029

[ref104] Sun H.; Chen G.; Wen B.; Sun J.; An H.; Pang J.; Xu W.; Yang X.; He S. Oligo-peptide ICF-6 inhibits hepatic stellate cell activation and ameliorates CCl4-induced liver fibrosis by suppressing NF-κB signaling and Wnt/β-catenin signaling. Journal of Pharmacological Sciences 2018, 136 (3), 133–141. 10.1016/j.jphs.2018.01.003. 29501581

[ref105] Ishigaki N.; Yamamoto N.; Jin H.; Uchida K.; Terai S.; Sakaida I. Continuos intravenous infusion of atrial natriuretic peptide (ANP) prevented liver fibrosis in rat. Biochem. Biophys. Res. Commun. 2009, 378 (3), 354–359. 10.1016/j.bbrc.2008.10.154. 18996092

[ref106] Wang X.; Lv L.; Chen Y.; Chen J. A CD36 synthetic peptide inhibits silica-induced lung fibrosis in the mice. Toxicol. Ind. Health 2010, 26 (1), 47–53. 10.1177/0748233709359274. 20056742

[ref107] Chen Y.; Wang X.; Weng D.; Tian L.; Lv L.; Tao S.; Chen J. A TSP-1 synthetic peptide inhibits bleomycin-induced lung fibrosis in mice. Experimental and Toxicologic Pathology 2009, 61 (1), 59–65. 10.1016/j.etp.2008.04.010. 18579356

[ref108] Murakami S.; Nagaya N.; Itoh T.; Fujii T.; Iwase T.; Hamada K.; Kimura H.; Kangawa K. C-type natriuretic peptide attenuates bleomycin-induced pulmonary fibrosis in mice. American Journal of Physiology-Lung Cellular and Molecular Physiology 2004, 287 (6), L1172–L1177. 10.1152/ajplung.00087.2004. 15285999

[ref109] Zhang M.; Yan Z.; Bu L.; An C.; Wang D.; Liu X.; Zhang J.; Yang W.; Deng B.; Xie J.; et al. Rapeseed protein-derived antioxidant peptide RAP alleviates renal fibrosis through MAPK/NF-κB signaling pathways in diabetic nephropathy. Drug Des., Dev. Ther. 2018, 12, 1255–1268. 10.2147/DDDT.S162288. PMC5958891 29795979

[ref110] Yuan Q.; Ren Q.; Li L.; Tan H.; Lu M.; Tian Y.; Huang L.; Zhao B.; Fu H.; Hou F. F.; et al. A Klotho-derived peptide protects against kidney fibrosis by targeting TGF-β signaling. Nat. Commun. 2022, 13, 438 10.1038/s41467-022-28096-z. 35064106 PMC8782923

[ref111] Guidotti G.; Brambilla L.; Rossi D. Cell-penetrating peptides: from basic research to clinics. Trends in pharmacological sciences 2017, 38 (4), 406–424. 10.1016/j.tips.2017.01.003. 28209404

[ref112] Joliot A.; Pernelle C.; Deagostini-Bazin H.; Prochiantz A. Antennapedia homeobox peptide regulates neural morphogenesis. Proc. Natl. Acad. Sci. U. S. A. 1991, 88 (5), 1864–1868. 10.1073/pnas.88.5.1864. 1672046 PMC51126

[ref113] Heitz F.; Morris M. C.; Divita G. Twenty years of cell-penetrating peptides: from molecular mechanisms to therapeutics. British journal of pharmacology 2009, 157 (2), 195–206. 10.1111/j.1476-5381.2009.00057.x. 19309362 PMC2697800

[ref114] Copolovici D. M.; Langel K.; Eriste E.; Langel U. Cell-penetrating peptides: design, synthesis, and applications. ACS Nano 2014, 8 (3), 1972–1994. 10.1021/nn4057269. 24559246

[ref115] Wang G. Post-translational modifications of natural antimicrobial peptides and strategies for peptide engineering. Curr. Biotechnol. 2012, 1 (1), 72–79. 10.2174/2211550111201010072. 24511461 PMC3914544

[ref116] Karandur D.; Wong K.-Y.; Pettitt B. M. Solubility and aggregation of Gly5 in water. J. Phys. Chem. B 2014, 118 (32), 9565–9572. 10.1021/jp503358n. 25019618 PMC4136715

[ref117] Guan S.; Munder A.; Hedtfeld S.; Braubach P.; Glage S.; Zhang L.; Lienenklaus S.; Schultze A.; Hasenpusch G.; Garrels W.; et al. Self-assembled peptide–poloxamine nanoparticles enable in vitro and in vivo genome restoration for cystic fibrosis. Nat. Nanotechnol. 2019, 14 (3), 287–297. 10.1038/s41565-018-0358-x. 30692673

[ref118] Lei J.; Sun L.; Huang S.; Zhu C.; Li P.; He J.; Mackey V.; Coy D. H.; He Q. The antimicrobial peptides and their potential clinical applications. Am. J. Transl. Res. 2019, 11 (7), 3919. 31396309 PMC6684887

[ref119] Luo Y.; Song Y. Mechanism of antimicrobial peptides: antimicrobial, anti-inflammatory and antibiofilm activities. International journal of molecular sciences 2021, 22 (21), 11401 10.3390/ijms222111401. 34768832 PMC8584040

[ref120] Batoni G.; Maisetta G.; Lisa Brancatisano F.; Esin S.; Campa M. Use of antimicrobial peptides against microbial biofilms: advantages and limits. Curr. Med. Chem. 2011, 18 (2), 256–279. 10.2174/092986711794088399. 21110801

[ref121] Pereira A. V.; de Barros G.; Pinto E. G.; Tempone A. G.; Orsi R. d. O.; dos Santos L. D.; Calvi S.; Ferreira R. S.; Pimenta D. C.; Barraviera B. Melittin induces in vitro death of Leishmania (Leishmania) infantum by triggering the cellular innate immune response. J. Venom. Anim. Toxins Incl. Trop. Dis. 2016, 10.1186/s40409-016-0055-x. PMC4706697 26752985

[ref122] Raghuraman H.; Chattopadhyay A. Melittin: a membrane-active peptide with diverse functions. Bioscience reports 2007, 27 (4–5), 189–223. 10.1007/s10540-006-9030-z. 17139559

[ref123] Papo N.; Shai Y. Can we predict biological activity of antimicrobial peptides from their interactions with model phospholipid membranes?. Peptides 2003, 24 (11), 1693–1703. 10.1016/j.peptides.2003.09.013. 15019200

[ref124] Pérez-Cordero J. J.; Lozano J. M.; Cortés J.; Delgado G. Leishmanicidal activity of synthetic antimicrobial peptides in an infection model with human dendritic cells. Peptides 2011, 32 (4), 683–690. 10.1016/j.peptides.2011.01.011. 21262294

[ref125] Mahdavi Abhari F.; Pirestani M.; Dalimi A. Anti-amoebic activity of a cecropin-melittin hybrid peptide (CM11) against trophozoites of Entamoeba histolytica. Wiener klinische Wochenschrift 2019, 131, 427–434. 10.1007/s00508-019-01540-9. 31451929

[ref126] Brady D.; Grapputo A.; Romoli O.; Sandrelli F. Insect cecropins, antimicrobial peptides with potential therapeutic applications. International journal of molecular sciences 2019, 20 (23), 5862 10.3390/ijms20235862. 31766730 PMC6929098

[ref127] Hong S.-M.; Kusakabe T.; Lee J.-M.; Tatsuke T.; Kawaguchi Y.; Kang M.-W.; Kang S.-W.; Kim K.-A.; Nho S.-K. Structure and expression analysis of the cecropin-E gene from the silkworm, Bombyx mori. Bioscience, biotechnology, and biochemistry 2008, 72 (8), 1992–1998. 10.1271/bbb.80082. 18685215

[ref128] Yi H.-Y.; Chowdhury M.; Huang Y.-D.; Yu X.-Q. Insect antimicrobial peptides and their applications. Applied microbiology and biotechnology 2014, 98, 5807–5822. 10.1007/s00253-014-5792-6. 24811407 PMC4083081

[ref129] Dhanjal D. S.; Chopra C.; Bhardwaj S.; Sharma P.; Nepovimova E.; Singh R.; Kuca K. Insect peptides with antimicrobial effects. In Antimicrobial Peptides; Ajesh K., Sreejith K., Eds.; Elsevier, 2023; pp 117–138.

[ref130] Akuffo H.; Hultmark D.; Engstöm A.; Frohlich D.; Kimbrell D. Drosophila antibacterial protein, cecropin A, differentially affects non-bacterial organisms such as Leishmania in a manner different from other amphipathic peptides. Int. J. Mol. Med. 1998, 1 (1), 77–159. 10.3892/ijmm.1.1.77. 9852202

[ref131] Kościuczuk E. M.; Lisowski P.; Jarczak J.; Strzałkowska N.; Jóźwik A.; Horbańczuk J.; Krzyżewski J.; Zwierzchowski L.; Bagnicka E. Cathelicidins: family of antimicrobial peptides. A review. Molecular biology reports 2012, 39, 10957–10970. 10.1007/s11033-012-1997-x. 23065264 PMC3487008

[ref132] Gallo R. L.; Kim K. J.; Bernfield M.; Kozak C. A.; Zanetti M.; Merluzzi L.; Gennaro R. Identification of CRAMP: A Cathelin Related Antimicrobial Peptide Expressed in the Embryonic and Adult Mouse (1). J. Biol. Chem. 1997, 272 (20), 13088–13093. 10.1074/jbc.272.20.13088. 9148921

[ref133] Marr A. K.; Cen S.; Hancock R. E. W.; McMaster W. R. Identification of synthetic and natural host defense peptides with leishmanicidal activity. Antimicrob. Agents Chemother. 2016, 60 (4), 2484–2491. 10.1128/AAC.02328-15. 26883699 PMC4808210

[ref134] Lynn M. A.; Kindrachuk J.; Marr A. K.; Jenssen H.; Panté N.; Elliott M. R.; Napper S.; Hancock R. E.; McMaster W. R. Effect of BMAP-28 antimicrobial peptides on Leishmania major promastigote and amastigote growth: role of leishmanolysin in parasite survival. PLoS neglected tropical diseases 2011, 5 (5), e1141 10.1371/journal.pntd.0001141. 21655347 PMC3104953

[ref135] Meade K. G.; O’Farrelly C. β-Defensins: farming the microbiome for homeostasis and health. Front. Immunol. 2019, 9, 3072 10.3389/fimmu.2018.03072. 30761155 PMC6362941

[ref136] Wilson S. S.; Wiens M. E.; Smith J. G. Antiviral mechanisms of human defensins. Journal of molecular biology 2013, 425 (24), 4965–4980. 10.1016/j.jmb.2013.09.038. 24095897 PMC3842434

[ref137] Holly M. K.; Diaz K.; Smith J. G. Defensins in viral infection and pathogenesis. Annual review of virology 2017, 4 (1), 369–391. 10.1146/annurev-virology-101416-041734. 28715972

[ref138] Kerenga B. K.; McKenna J. A.; Harvey P. J.; Quimbar P.; Garcia-Ceron D.; Lay F. T.; Phan T. K.; Veneer P. K.; Vasa S.; Parisi K.; et al. Salt-tolerant antifungal and antibacterial activities of the corn defensin ZmD32. Front. Microbiol. 2019, 10, 795 10.3389/fmicb.2019.00795. 31031739 PMC6474387

[ref139] Polesello V.; Segat L.; Crovella S.; Zupin L. Candida infections and human defensins. Protein Peptide Lett. 2017, 24 (8), 747–756. 10.2174/0929866524666170807125245. 28782479

[ref140] Yoo Y.-J.; Kwon I.; Oh S.-R.; Perinpanayagam H.; Lim S.-M.; Ahn K.-B.; Lee Y.; Han S.-H.; Chang S.-W.; Baek S.-H.; et al. Antifungal effects of synthetic human Beta-defensin-3-c15 peptide on Candida Albicans–infected root dentin. J. Endodontics 2017, 43 (11), 1857–1861. 10.1016/j.joen.2017.06.035. 28951032

[ref141] Dong H.; Lv Y.; Zhao D.; Barrow P.; Zhou X. Defensins: the case for their use against mycobacterial infections. J. Immunol. Res. 2016, 2016, 7515687 10.1155/2016/7515687. 27725944 PMC5048032

[ref142] Souza G. S.; de Carvalho L. P.; de Melo E. J. T.; da Silva F. C. V.; Machado O. L. T.; Gomes V. M.; de Oliveira Carvalho A. A synthetic peptide derived of the β 2−β 3 loop of the plant defensin from Vigna unguiculata seeds induces Leishmania amazonensis apoptosis-like cell death. Amino Acids 2019, 51, 1633–1648. 10.1007/s00726-019-02800-8. 31654210

[ref143] Pincus M. R. Physiological structure and function of proteins. In Cell physiology source book, 3rd ed.; Sperelakis N., Ed.; Elsevier: 2001; pp 19–42.

[ref144] Pino-Angeles A.; Leveritt J. M. III; Lazaridis T. Pore structure and synergy in antimicrobial peptides of the magainin family. PLoS Comput. Biol. 2016, 12 (1), e1004570 10.1371/journal.pcbi.1004570. 26727376 PMC4699650

[ref145] Bagheri M. Aggregation vs. Fusion of Negatively Charged Lipid Bilayers Induced by Bactenecin and Magainin Derivatives. Biophys. J. 2018, 114 (3), 453a 10.1016/j.bpj.2017.11.2504.

[ref146] Hasan M.; Karal M. A. S.; Levadnyy V.; Yamazaki M. Mechanism of initial stage of pore formation induced by antimicrobial peptide magainin 2. Langmuir 2018, 34 (10), 3349–3362. 10.1021/acs.langmuir.7b04219. 29446954

[ref147] Patocka J.; Nepovimova E.; Klimova B.; Wu Q.; Kuca K. Antimicrobial peptides: amphibian host defense peptides. Curr. Med. Chem. 2019, 26 (32), 5924–5946. 10.2174/0929867325666180713125314. 30009702

[ref148] Nyström L.; Malmsten M. Membrane interactions and cell selectivity of amphiphilic anticancer peptides. Curr. Opin. Colloid Interface Sci. 2018, 38, 1–17. 10.1016/j.cocis.2018.06.009.

[ref149] Bacalum M.; Radu M. Cationic antimicrobial peptides cytotoxicity on mammalian cells: an analysis using therapeutic index integrative concept. International Journal of Peptide Research and Therapeutics 2015, 21, 47–55. 10.1007/s10989-014-9430-z.

[ref150] Guerrero E.; Saugar J. M.; Matsuzaki K.; Rivas L. Role of positional hydrophobicity in the leishmanicidal activity of magainin 2. Antimicrob. Agents Chemother. 2004, 48 (8), 2980–2986. 10.1128/AAC.48.8.2980-2986.2004. 15273109 PMC478506

[ref151] Giacometti A.; Cirioni O.; Del Prete M. S.; Barchiesi F.; Scalise G. Short-term exposure to membrane-active antibiotics inhibits Cryptosporidium parvum infection in cell culture. Antimicrob. Agents Chemother. 2000, 44 (12), 3473–3475. 10.1128/AAC.44.12.3473-3475.2000. 11083662 PMC90227

[ref152] Feder R.; Dagan A.; Mor A. Structure-activity relationship study of antimicrobial dermaseptin S4 showing the consequences of peptide oligomerization on selective cytotoxicity. J. Biol. Chem. 2000, 275 (6), 4230–4238. 10.1074/jbc.275.6.4230. 10660589

[ref153] Chaves R. X.; Quelemes P. V.; Leite L. M.; Aquino D. S.A.; Amorim L. V.; Rodrigues K. A.F.; Campelo Y. D.M.; Veras L. M.C; Bemquerer M. P.; Ramos-Jesus J.; et al. Antileishmanial and immunomodulatory effects of Dermaseptin-01, a promising peptide against Leishmania amazonensis. Curr. Bioactive Compounds 2017, 13 (4), 305–311. 10.2174/1573407212666161014131415.

[ref154] Konno K.; Hisada M.; Naoki H.; Itagaki Y.; Fontana R.; Rangel M.; Oliveira J. S.; dos Santos Cabrera M. P.; Neto J. R.; Hide I. Eumenitin, a novel antimicrobial peptide from the venom of the solitary eumenine wasp Eumenes rubronotatus. Peptides 2006, 27 (11), 2624–2631. 10.1016/j.peptides.2006.04.013. 16762455

[ref155] Sabia Junior E. F.; Menezes L. F. S.; de Araújo I. F. S.; Schwartz E. F. Natural occurrence in venomous arthropods of antimicrobial peptides active against protozoan parasites. Toxins 2019, 11 (10), 563 10.3390/toxins11100563. 31557900 PMC6832604

[ref156] Rangel M.; dos Santos Cabrera M. P.; Kazuma K.; Ando K.; Wang X.; Kato M.; Nihei K.-i.; Hirata I. Y.; Cross T. J.; Garcia A. N. Chemical and biological characterization of four new linear cationic α-helical peptides from the venoms of two solitary eumenine wasps. Toxicon 2011, 57 (7–8), 1081–1092. 10.1016/j.toxicon.2011.04.014. 21549739

[ref157] Dale B. A.; Fredericks L. P. Antimicrobial peptides in the oral environment: expression and function in health and disease. Curr Issues Mol. Biol. 2005, 7 (2), 119–134. 10.1093/jac/dki103. 16053246 PMC1479858

[ref158] Khurshid Z.; Najeeb S.; Mali M.; Moin S. F.; Raza S. Q.; Zohaib S.; Sefat F.; Zafar M. S. Histatin peptides: Pharmacological functions and their applications in dentistry. Saudi Pharmaceutical Journal 2017, 25 (1), 25–31. 10.1016/j.jsps.2016.04.027. 28223859 PMC5310145

[ref159] Luque-Ortega J. R.; van’t Hof W.; Veerman E. C. I.; Saugar J. M.; Rivas L. Human antimicrobial peptide histatin 5 is a cell-penetrating peptide targeting mitochondrial ATP synthesis in Leishmania. FASEB J. 2008, 22 (6), 1817–1828. 10.1096/fj.07-096081. 18230684

[ref160] Lee Y.-C. J.; Shirkey J. D.; Park J.; Bisht K.; Cowan A. J. An overview of antiviral peptides and rational biodesign considerations. BioDesign Res. 2022, 2022, 9898241 10.34133/2022/9898241. PMC10521750 37850133

[ref161] Dhingra N.; Bhardwaj R.; Bhardwaj U.; Kapoor K. Design of hACE2-based small peptide inhibitors against spike protein of SARS-CoV-2: a computational approach. Structural Chemistry 2023, 34 (5), 1843–1856. 10.1007/s11224-023-02125-z. PMC9875775 36714014

[ref162] Zhang G.; Pomplun S.; Loftis A. R.; Tan X.; Loas A.; Pentelute B. L. Investigation of ACE2 N-terminal fragments binding to SARS-CoV-2 Spike RBD. BioRxiv 2020, 10.1101/2020.03.19.999318.

[ref163] Xia S.; Yan L.; Xu W.; Agrawal A. S.; Algaissi A.; Tseng C.-T. K.; Wang Q.; Du L.; Tan W.; Wilson I. A.; et al. A pan-coronavirus fusion inhibitor targeting the HR1 domain of human coronavirus spike. Sci. Adv. 2019, 5 (4), eaav4580 10.1126/sciadv.aav4580. 30989115 PMC6457931

[ref164] Xia S.; Zhu Y.; Liu M.; Lan Q.; Xu W.; Wu Y.; Ying T.; Liu S.; Shi Z.; Jiang S.; et al. Fusion mechanism of 2019-nCoV and fusion inhibitors targeting HR1 domain in spike protein. Cell. Mol. Immunol. 2020, 17 (7), 765–767. 10.1038/s41423-020-0374-2. 32047258 PMC7075278

[ref165] Xia S.; Liu M.; Wang C.; Xu W.; Lan Q.; Feng S.; Qi F.; Bao L.; Du L.; Liu S.; et al. Inhibition of SARS-CoV-2 (previously 2019-nCoV) infection by a highly potent pan-coronavirus fusion inhibitor targeting its spike protein that harbors a high capacity to mediate membrane fusion. Cell Res. 2020, 30 (4), 343–355. 10.1038/s41422-020-0305-x. 32231345 PMC7104723

[ref166] Struck A.-W.; Axmann M.; Pfefferle S.; Drosten C.; Meyer B. A hexapeptide of the receptor-binding domain of SARS corona virus spike protein blocks viral entry into host cells via the human receptor ACE2. Antiviral research 2012, 94 (3), 288–296. 10.1016/j.antiviral.2011.12.012. 22265858 PMC7114193

[ref167] Wang Q.; Zhang Y.; Wu L.; Niu S.; Song C.; Zhang Z.; Lu G.; Qiao C.; Hu Y.; Yuen K.-Y.; et al. Structural and functional basis of SARS-CoV-2 entry by using human ACE2. Cell 2020, 181 (4), 894–904.e9. 10.1016/j.cell.2020.03.045. 32275855 PMC7144619

[ref168] Bestle D.; Heindl M. R.; Limburg H.; Van Lam van T.; Pilgram O.; Moulton H.; Stein D. A; Hardes K.; Eickmann M.; Dolnik O. TMPRSS2 and furin are both essential for proteolytic activation of SARS-CoV-2 in human airway cells. Life Sci. Alliance 2020, 3 (9), e202000786 10.26508/lsa.202000786. 32703818 PMC7383062

[ref169] Zhao H.; Zhou J.; Zhang K.; Chu H.; Liu D.; Poon V. K.-M.; Chan C. C.-S.; Leung H.-C.; Fai N.; Lin Y.-P.; et al. A novel peptide with potent and broad-spectrum antiviral activities against multiple respiratory viruses. Sci. Rep 2016, 6, 22008 10.1038/srep22008. 26911565 PMC4766503

[ref170] Petrova V. N.; Russell C. A. The evolution of seasonal influenza viruses. Nature Reviews Microbiology 2018, 16 (1), 47–60. 10.1038/nrmicro.2017.118. 29081496

[ref171] Agamennone M.; Fantacuzzi M.; Vivenzio G.; Scala M. C.; Campiglia P.; Superti F.; Sala M. Antiviral peptides as anti-influenza agents. International Journal of Molecular Sciences 2022, 23 (19), 11433 10.3390/ijms231911433. 36232735 PMC9569631

[ref172] Sauter N. K.; Hanson J. E.; Glick G. D.; Brown J. H.; Crowther R. L.; Park S. J.; Skehel J. J.; Wiley D. C. Binding of influenza virus hemagglutinin to analogs of its cell-surface receptor, sialic acid: analysis by proton nuclear magnetic resonance spectroscopy and X-ray crystallography. Biochemistry 1992, 31 (40), 9609–9621. 10.1021/bi00155a013. 1327122

[ref173] Skehel J. J.; Bizebard T.; Bullough P. A.; Hughson F. M.; Knossow M.; Steinhauer D. A.; Warton S. A.; Wiley D. C. Membrane fusion by influenza hemagglutinin. Cold Spring Harb. Symp. Quant. Biol. 1995, 60, 573–580. 10.1101/SQB.1995.060.01.061. 8824430

[ref174] Jones J. C.; Turpin E. A.; Bultmann H.; Brandt C. R.; Schultz-Cherry S. Inhibition of influenza virus infection by a novel antiviral peptide that targets viral attachment to cells. Journal of virology 2006, 80 (24), 11960–11967. 10.1128/JVI.01678-06. 17005658 PMC1676284

[ref175] Jones J. C.; Settles E. W.; Brandt C. R.; Schultz-Cherry S. Identification of the minimal active sequence of an anti-influenza virus peptide. Antimicrob. Agents Chemother. 2011, 55 (4), 1810–1813. 10.1128/AAC.01428-10. 21220525 PMC3067171

[ref176] Matsubara T.; Sumi M.; Kubota H.; Taki T.; Okahata Y.; Sato T. Inhibition of influenza virus infections by sialylgalactose-binding peptides selected from a phage library. Journal of medicinal chemistry 2009, 52 (14), 4247–4256. 10.1021/jm801570y. 19558186

[ref177] Pietrantoni A.; Dofrelli E.; Tinari A.; Ammendolia M. G.; Puzelli S.; Fabiani C.; Donatelli I.; Superti F. Bovine lactoferrin inhibits influenza A virus induced programmed cell death in vitro. Biometals 2010, 23, 465–475. 10.1007/s10534-010-9323-3. 20232110

[ref178] Ammendolia M. G.; Agamennone M.; Pietrantoni A.; Lannutti F.; Siciliano R. A.; De Giulio B.; Amici C.; Superti F. Bovine lactoferrin-derived peptides as novel broad-spectrum inhibitors of influenza virus. Pathogens and global health 2012, 106 (1), 12–19. 10.1179/2047773212Y.0000000004. 22595270 PMC4001507

[ref179] Scala M. C.; Agamennone M.; Pietrantoni A.; Di Sarno V.; Bertamino A.; Superti F.; Campiglia P.; Sala M. Discovery of a novel tetrapeptide against influenza a virus: Rational design, synthesis, bioactivity evaluation and computational studies. Pharmaceuticals 2021, 14 (10), 959 10.3390/ph14100959. 34681184 PMC8537277

[ref180] Tambunan U. S. F.; Amri N.; Parikesit A. A. In silico design of cyclic peptides as influenza virus, a subtype H1N1 neuraminidase inhibitor. Aft. J. Biotechnol. 2012, 11 (52), 11474–11491. 10.5897/AJB11.4094.

[ref181] Upadhyay A.; Chompoo J.; Taira N.; Fukuta M.; Gima S.; Tawata S. Solid-phase synthesis of mimosine tetrapeptides and their inhibitory activities on neuraminidase and tyrosinase. Journal of agricultural and food chemistry 2011, 59 (24), 12858–12863. 10.1021/jf203494t. 22047208

[ref182] Chen J.; Feng S.; Xu Y.; Huang X.; Zhang J.; Chen J.; An X.; Zhang Y.; Ning X. Discovery and characterization of a novel peptide inhibitor against influenza neuraminidase. RSC Medicinal Chemistry 2020, 11 (1), 148–154. 10.1039/C9MD00473D. 33479615 PMC7433756

[ref183] Poch O.; Sauvaget I.; Delarue M.; Tordo N. Identification of four conserved motifs among the RNA-dependent polymerase encoding elements. EMBO journal 1989, 8 (12), 3867–3874. 10.1002/j.1460-2075.1989.tb08565.x. 2555175 PMC402075

[ref184] Tiley L. S.; Hagen M.; Matthews J. T.; Krystal M. Sequence-specific binding of the influenza virus RNA polymerase to sequences located at the 5′ends of the viral RNAs. Journal of virology 1994, 68 (8), 5108–5116. 10.1128/jvi.68.8.5108-5116.1994. 8035510 PMC236454

[ref185] Mitra A. K.; Mawson A. R. Neglected tropical diseases: epidemiology and global burden. Tropical medicine and infectious disease 2017, 2 (3), 36 10.3390/tropicalmed2030036. 30270893 PMC6082091

[ref186] Bhatt S.; Gething P. W.; Brady O. J.; Messina J. P.; Farlow A. W.; Moyes C. L.; Drake J. M.; Brownstein J. S.; Hoen A. G.; Sankoh O.; et al. The global distribution and burden of dengue. Nature 2013, 496 (7446), 504–507. 10.1038/nature12060. 23563266 PMC3651993

[ref187] Pitcher T. J.; Sarathy V. V.; Matsui K.; Gromowski G. D.; Huang C. Y. H.; Barrett A. D. T. Functional analysis of dengue virus (DENV) type 2 envelope protein domain 3 type-specific and DENV complex-reactive critical epitope residues. Journal of General Virology 2015, 96 (2), 288–293. 10.1099/vir.0.070813-0. 25351518 PMC4298678

[ref188] Cui X.; Wu Y.; Fan D.; Gao N.; Ming Y.; Wang P.; An J. Peptides P4 and P7 derived from E protein inhibit entry of dengue virus serotype 2 via interacting with β3 integrin. Antiviral Res. 2018, 155, 20–27. 10.1016/j.antiviral.2018.04.018. 29709564

[ref189] Hrobowski Y. M.; Garry R. F.; Michael S. F. Peptide inhibitors of dengue virus and West Nile virus infectivity. Virol. J. 2005, 2, 49 10.1186/1743-422X-2-49. 15927084 PMC1177995

[ref190] Muller D. A.; Young P. R. The flavivirus NS1 protein: molecular and structural biology, immunology, role in pathogenesis and application as a diagnostic biomarker. Antiviral research 2013, 98 (2), 192–208. 10.1016/j.antiviral.2013.03.008. 23523765

[ref191] Lin T.-Y.; Chu C.; Chiu C.-H. Lactoferrin inhibits enterovirus 71 infection of human embryonal rhabdomyosarcoma cells in vitro. Journal of infectious diseases 2002, 186 (8), 1161–1164. 10.1086/343809. 12355368

[ref192] Uddin M. B.; Lee B.-H.; Nikapitiya C.; Kim J.-H.; Kim T.-H.; Lee H.-C.; Kim C. G.; Lee J.-S.; Kim C.-J. Inhibitory effects of bee venom and its components against viruses in vitro and in vivo. Journal of Microbiology 2016, 54, 853–866. 10.1007/s12275-016-6376-1. 27888461 PMC7091203

[ref193] Chen W.; Liu Z.; Zhang Q.; Yan Q.; Jing S. Induction and antiviral activity of human β-defensin 3 in intestinal cells with picornavirus infection. Acta Virol. 2018, 62 (3), 287 10.4149/av_2018_222. 30160144

[ref194] Tiwari V.; Liu J.; Valyi-Nagy T.; Shukla D. Anti-heparan sulfate peptides that block herpes simplex virus infection in vivo. J. Biol. Chem. 2011, 286 (28), 25406–25415. 10.1074/jbc.M110.201103. 21596749 PMC3137111

[ref195] Tan C. W.; Chan Y. F.; Sim K. M.; Tan E. L.; Poh C. L. Inhibition of enterovirus 71 (EV-71) infections by a novel antiviral peptide derived from EV-71 capsid protein VP1. PLoS One 2012, 7 (5), e34589 10.1371/journal.pone.0034589. 22563456 PMC3341398

